# Sex-biased genetic regulation of inflammatory proteins in the Dutch population

**DOI:** 10.1186/s12864-024-10065-z

**Published:** 2024-02-08

**Authors:** Collins K. Boahen, Hannah Abee, Isis Ricaño Ponce, Leo A. B. Joosten, Mihai G. Netea, Vinod Kumar

**Affiliations:** 1 Department of Internal Medicine and Radboud Institute of Molecular Life Sciences (RIMLS), Radboud University Medical Center, Nijmegen, 6525 HP the Netherlands; 2 Department of Internal Medicine and Radboud Center for Infectious Diseases (RCI), Radboud University Medical Center, Nijmegen, 6525 HP the Netherlands; 3 Department of Medical Genetics, Iuliu Hatieganu University of Medicine and Pharmacia, Cluj-Napoca-Napoca, Romania; 4 Department of Genetics, University of Groningen, University Medical Center Groningen, Groningen, 9700 RB the Netherlands; 5 Department for Immunology and Metabolism, Life and Medical Sciences Institute (LIMES), University of Bonn, Bonn, Germany; 6 Nitte (Deemed to Be University), Medical Sciences Complex, Nitte University Centre for Science Education and Research (NUCSER), Deralakatte, Mangalore, 575018 India

**Keywords:** Sex-stratified genetic analysis, Inflammation, pQTL, GWAS, Biomarkers

## Abstract

**Background:**

Significant differences in immune responses, prevalence or susceptibility of diseases and treatment responses have been described between males and females. Despite this, sex-differentiation analysis of the genetic architecture of inflammatory proteins is largely unexplored. We performed sex-stratified meta-analysis after protein quantitative trait loci (pQTL) mapping using inflammatory biomarkers profiled using targeted proteomics (Olink inflammatory panel) of two population-based cohorts of Europeans.

**Results:**

Even though, around 67% of the pQTLs demonstrated shared effect between sexes, colocalization analysis identified two loci in the males (*LINC01135* and *ITGAV*) and three loci (*CNOT10*, *SRD5A2*, and *LILRB5*) in the females with evidence of sex-dependent modulation by pQTL variants. Furthermore, we identified pathways with relevant functions in the sex-biased pQTL variants. We also showed through cross-validation that the sex-specific pQTLs are linked with sex-specific phenotypic traits.

**Conclusion:**

Our study demonstrates the relevance of genetic sex-stratified analysis in the context of genetic dissection of protein abundances among individuals and reveals that, sex-specific pQTLs might mediate sex-linked phenotypes. Identification of sex-specific pQTLs associated with sex-biased diseases can help realize the promise of individualized treatment.

**Supplementary Information:**

The online version contains supplementary material available at 10.1186/s12864-024-10065-z.

## Background

Differences in inflammation between males and females can explain sex-biased susceptibility and severity of various common diseases such as atherosclerosis, cancer, autoimmune diseases and many more [1]. While females are less prone to infectious diseases than males, they account for more than 80% of individuals presented with autoimmune diseases such as systemic lupus erythematosus (SLE), rheumatic arthritis (RA) and autoimmune thyroid disease [2]. Sex differences in immunological responses and disease susceptibility may be influenced by the complex interaction of sex hormones [3], host microbiome [4], immune-regulatory genes located on the X-chromosome [5] and environmental exposures [6–8]. It is also possible that genetic polymorphisms associated with immune phenotypes partially account for sex-based differences in immune response. In this regard and given the recent recognition of sex-stratified analysis, studies in the context of epigenetics [9, 10] and transcriptomics [11, 12] have been conducted. The disproportionate sex influence on all these phenotypes, including treatment efficacy [13], makes it imperative to understand the role of sex on modulation of inflammation in complex traits and diseases which will ultimately help our quest of developing personalized treatments.

Many of the key inflammatory markers which are released into the bloodstream during inflammation and are associated with chronic diseases are proteins, the functional molecules encoded by the genome [14]. A plethora of protein quantitative trait loci (pQTL) studies have been conducted to characterize genetic variants associated with circulating protein levels in both healthy and disease individuals [15–21]. Despite the burgeoning number of studies reporting sex-based differences in the immune response, most of the research on pQTLs analysis mainly adjust for sex differences in the model without seeking to identify sex-specific pQTLs. A recent study clearly demonstrated sex-dependent effect on the circulating concentrations of inflammatory proteins [22]. Therefore, identifying the precise factors driving the inter-individual variability in inflammatory protein levels especially in sex-dependent fashion will help to comprehensively understand sex differences in inflammatory-driven diseases and to make meaningful prediction of individual risk for diseases.

In the present study, using two population-based cohorts, we aimed to identify which genetic variants affect inflammatory protein levels in sex-specific manner. We identified and compared genetic variants associated with protein concentrations profiled with the Olink Inflammation panel using meta-analytic approach in males and females separately. We demonstrate that while the regulation of numerous pQTL variants is independent of sex, some key loci act discordantly between sexes and are correlated with sex-dependent traits.

## Results

**Fig. 1:**
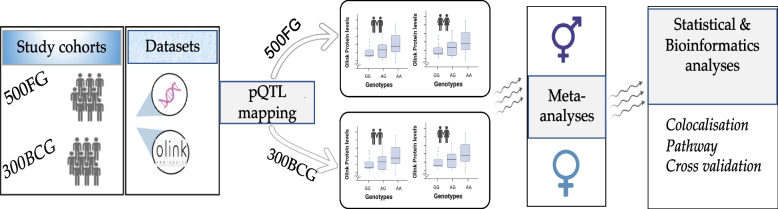
Graphical representation of study cohorts, design and analysis conducted. This study utilized two population-based cohorts (500FG & 300 BCG) of individuals of European decent. Imputed genetic data and plasma protein abundances profiled with the Olink inflammatory panel were available for protein quantitative trait (pQTL) mapping in sex-dependent fashion. The resulting summary statistics were integrated using the meta-analytic approach for males and females separately. Colocalization and functional enrichment analysis of the identified meta-analyzed pQTL variants were conducted. Finally, we cross-validated the identified pQTL variants with previously published molecular traits

We aimed to identify single nucleotide polymorphisms (SNPs) associated with plasma protein concentrations profiled in males and females samples separately using two different population-based cohorts. To enhance statistical power and to detect robust signals, pQTL association results of 66 inflammatory proteins and 4,028,465 SNPs of both cohorts were integrated using meta-analysis. A general overview of the cohorts and analyses conducted is displayed in Fig. 1.

### Identification of pQTLs in males

**Fig. 2:**
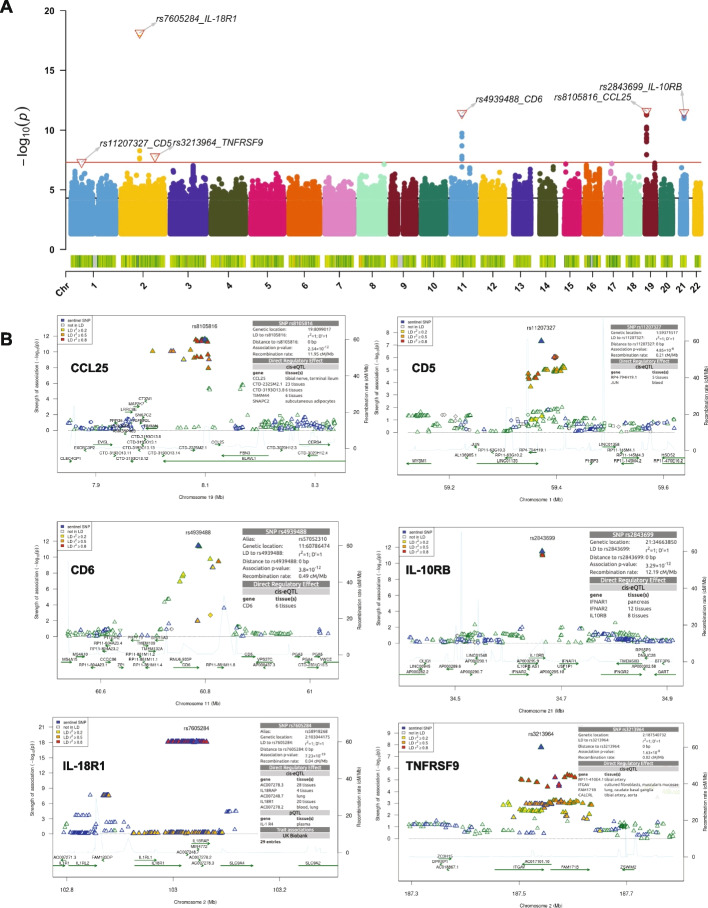
Summary of pQTL meta-analysis results in males. **A** Manhattan plot depicting the association results of significant genetic variants (*P* < 0.05). The red bold horizontal line marks the genome-wide significant threshold (*p*-value < 1 × 10^–8^) and the black dashed denotes the suggestive threshold (*p*-value = 5 × 10^–5^). Top pQTL variants and their correlated proteins are displayed on the plot. **B** Regional association plots of the genome-wide significant loci (*p* < 1 × 10^–8^). The -log10 association *p*-values are plotted on the y-axis against physical position (NCBI build 36) of each marker on the x-axis. The pQTL variants are color coded according to their correlation coefficient (r^2^) with the top SNP using the hg19/1000 Genomes European samples

In the males (*N* = 318), we observed a total of six genome-wide significant pQTLs (*P* < 5 × 10^–8^) which comprises of two *trans*-pQTL variants (rs3213964 and rs11207327) regulating TNFRSF9 and CD5 levels respectively. The four *cis-acting* pQTL variants (lying 250 kb window around the tested protein-coding gene) were associated with CCL25, CD6, IL-10RB and IL-18R1 proteins. Summary of the meta-analyzed results are represented with Manhattan plot (Fig. 2A) while regional association plots are used to zoom into the genomic regions surrounding the index pQTL variants of each significant pQTL locus (Fig. 2B). In general, the *cis-acting* pQTL variants showed stronger genetic associations. For example, the most strongly associated intronic SNP rs7605284 with IL-18R1 (*P* = 7.22 × 10^–19^,Zscore = 8.871) followed by SNP rs2843699 correlating with IL-10RB (*P* = 3.29 × 10^–12^, Zscore = -6.965) were all residing in the *cis*-regions. Interestingly, *trans*-pQTL variant (rs11207327) correlating with CD5 levels resides in the *LINC01135* locus, suggesting that genetic polymorphisms in both the protein-coding and non-coding regions play a role in regulating variations in plasma protein levels. The most significantly associated genetic variant for each protein in the males is reported in Table 1. Detailed summary statistics for the results of pQTL mapping in each cohort and meta-analyzed results with suggestive associations for the males are presented in Table S1.

**Table 1:** Summary statistics of index-pQTLs for males after meta-analysis

MarkerName	A1	A2	Weight	Zscore	P.value	Direction	chr	pos	Protein
**rs7605284**	**t**	**g**	**318**	**8.871**	**7.23E-19**	** + + **	**2**	**103044175**	**IL.18R1**
**rs8105816**	**t**	**c**	**318**	**7.001**	**2.54E-12**	** + + **	**19**	**8099017**	**CCL25**
**rs2843699**	**t**	**c**	**318**	**-6.965**	**3.29E-12**	**–**	**21**	**34663850**	**IL.10RB**
**rs4939488**	**t**	**c**	**318**	**6.944**	**3.80E-12**	** + + **	**11**	**60786474**	**CD6**
**rs3213964**	**a**	**g**	**318**	**-5.647**	**1.63E-08**	**–**	**2**	**187540732**	**TNFRSF9**
**rs11207327**	**a**	**g**	**318**	**-5.457**	**4.85E-08**	**–**	**1**	**59371517**	**CD5**
rs7259738	c	g	318	-5.425	5.79E-08	–	19	53308330	CD40
rs2041190	a	g	318	5.407	6.42E-08	+ +	17	32634541	MCP.2
rs3098547	a	g	318	5.395	6.86E-08	+ +	15	27873562	HGF
rs9771768	a	g	318	5.392	6.98E-08	+ +	8	139165953	IL7
rs1986471	t	c	318	5.337	9.43E-08	+ +	3	127001452	OPG
rs9323902	t	g	318	-5.296	1.19E-07	–	14	94572110	LAP.TGF.beta.1
rs9983324	a	g	318	-5.264	1.41E-07	–	21	21130809	CXCL6
rs678815	c	g	318	-5.258	1.46E-07	–	11	102713777	MMP.1
rs72800254	a	g	318	-5.224	1.75E-07	–	16	83201614	TNFRSF14
rs58814158	t	g	318	-5.208	1.91E-07	–	13	110820255	FGF.23
rs924935	a	g	318	5.161	2.45E-07	+ +	3	16172032	CCL28
rs7530589	a	g	318	-5.134	2.83E-07	–	1	22756991	TRANCE
rs2346994	a	g	318	5.109	3.24E-07	+ +	16	26478953	CCL23
rs7429951	a	g	318	-5.104	3.33E-07	–	3	18031227	uPA
rs12578430	t	c	318	5.096	3.47E-07	+ +	12	104093212	CDCP1
rs72783206	a	g	318	-5.067	4.03E-07	–	2	34173056	IL.17A
rs7151919	a	g	318	-5.049	4.43E-07	–	14	21139505	CSF.1
rs1963248	t	g	318	5.04	4.65E-07	+ +	1	74346006	FGF.19
rs112953000	t	c	318	-5.01	5.45E-07	–	21	37365315	MCP.4
rs2604330	a	g	318	4.994	5.92E-07	+ +	8	15729358	TNFB
rs2568222	a	g	318	4.987	6.12E-07	+ +	2	85377979	PD.L1
rs73232950	c	g	318	4.981	6.32E-07	+ +	21	46208128	CASP.8
rs1233829	a	t	318	4.972	6.62E-07	+ +	1	198041374	CXCL11
rs10977778	c	g	318	-4.97	6.69E-07	–	9	9459604	CXCL10
rs2543063	t	c	318	4.954	7.27E-07	+ +	8	39832166	Flt3L
rs112770619	a	g	318	-4.942	7.73E-07	–	8	119484547	TWEAK
rs4942873	t	c	318	4.941	7.76E-07	+ +	13	50392047	IL6
rs60844779	a	t	318	4.925	8.45E-07	+ +	5	149456772	CCL19
rs2165385	t	c	318	-4.924	8.46E-07	–	18	27371049	CST5
rs4854604	a	c	318	4.924	8.50E-07	+ +	3	133780263	CD8A
rs34011530	c	g	318	4.894	9.87E-07	+ +	11	1689461	NT.3
rs2552275	c	g	318	4.892	9.96E-07	+ +	8	6022581	CD244
rs9324481	c	g	318	-4.887	1.03E-06	–	8	139121726	CXCL1
rs9502959	t	c	318	-4.877	1.08E-06	–	6	206599	TRAIL
rs1814451	t	c	318	4.876	1.09E-06	+ +	17	47540123	DNER
rs72977655	t	c	318	-4.867	1.13E-06	–	2	235708696	EN.RAGE
rs4335544	a	c	318	4.854	1.21E-06	+ +	11	21,330,182	ADA
rs2570672	c	g	318	4.85	1.24E-06	+ +	8	6026307	IL18
rs75674858	a	g	318	4.847	1.25E-06	+ +	4	44388139	IL8
rs10933215	t	c	318	4.843	1.28E-06	+ +	2	228661357	IL10
rs4328681	a	g	318	4.833	1.34E-06	+ +	2	45261612	MMP.10
rs357292	t	g	318	4.79	1.67E-06	+ +	5	38923732	OSM
rs493480	c	g	318	4.777	1.78E-06	+ +	15	55879574	CXCL9
rs9855230	a	g	318	4.751	2.03E-06	+ +	3	64642056	CCL4
rs4752902	a	g	318	4.742	2.12E-06	+ +	11	48148779	LIF.R
rs11017041	a	c	318	-4.741	2.12E-06	–	10	131795756	TGF.alpha
rs1034527	t	c	318	-4.738	2.16E-06	–	8	120939125	SCF
rs744768	a	g	318	4.732	2.22E-06	+ +	7	132014951	CCL3
rs10756341	t	c	318	4.725	2.31E-06	+ +	9	12203051	ST1A1
rs1216465	a	c	318	4.723	2.33E-06	+ +	11	100343360	MCP.1
rs11881877	a	g	318	-4.712	2.46E-06	–	19	53401760	IL.12B
rs57254266	a	g	318	4.711	2.47E-06	+ +	11	5352458	VEGFA
rs9771768	a	g	318	4.704	2.55E-06	+ +	8	139165953	CXCL5
rs4839524	a	g	318	-4.704	2.55E-06	–	1	116800201	X4E.BP1
rs167500	a	c	318	4.684	2.81E-06	+ +	18	1907316	SIRT2
rs85023	a	g	318	4.623	3.78E-06	+ +	20	45181296	CX3CL1
rs1759325	t	c	318	-4.616	3.91E-06	–	10	59838496	CCL11
rs7964436	t	c	318	4.581	4.64E-06	+ +	12	15561776	CCL20
rs7628951	a	g	318	4.562	5.06E-06	+ +	3	56457705	AXIN1
rs8060853	a	g	318	4.553	5.29E-06	+ +	16	30403858	FGF.21

### Identification of pQTLs in females

**Fig. 3:**
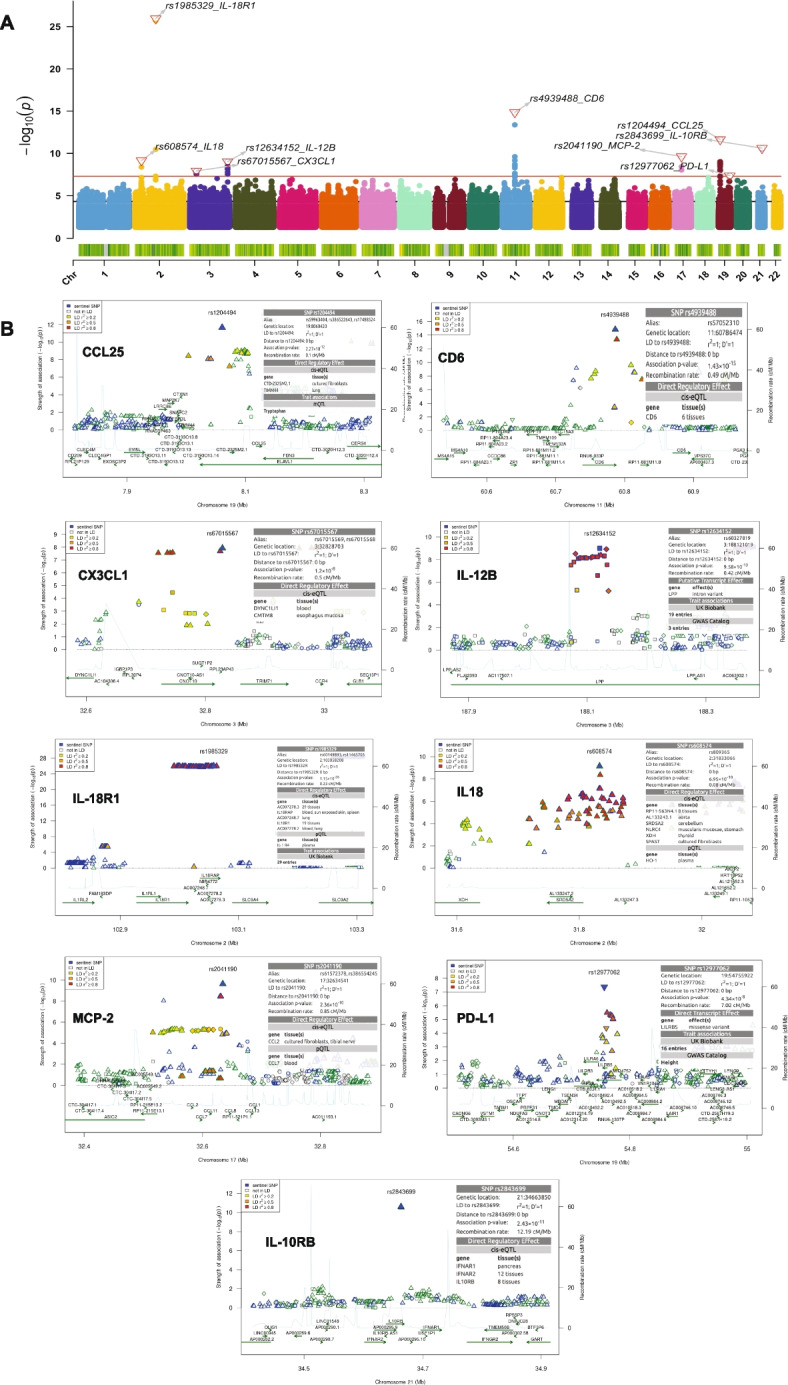
Summary of pQTL meta-analysis results in females. **A** Manhattan plot illustrating the association results of significant genetic variants (*P* < 0.05). The red bold horizontal line denotes the genome-wide significant threshold (*p*-value < 1 × 10^–8^) and the black dashed denotes the suggestive threshold (*p*-value = 5 × 10^–5^). Top pQTL variants and their correlated proteins are displayed on the plot. **B** Regional association plots of the genome-wide significant loci (*p* < 1 × 10^–8^). The -log10 association *p*-values are plotted on the y-axis against physical position (NCBI build 36) of each marker on the x-axis. The pQTL variants are color coded according to their correlation coefficient (r^2^) with the top SNP using the hg19/1000 Genomes European samples

Next, we repeated the meta-analysis of the 66 proteins in the females (*N* = 359) to uncover SNPs controlling inflammatory protein levels in females. We identified a total of nine genome-wide significant pQTLs comprising four *trans* pQTL variants (rs12634152, rs608574, rs12977062 and rs67015567) which affected IL-12B, IL18, PD-L1 and CX3CL1 abundances respectively. The five *cis*-acting pQTL variants were associated with proteins such as IL-18R1, MCP-2, CCL25, CD6, and IL-10RB. The SNP rs1985329 showing the most significant association (*P* = 1.15 × 10^–26^) is within an intron mapping to *IL18R1*. All the 9 pQTLs showed stronger associations in females than in males except rs2843699-IL-10RB association (*P* = 2.43 × 10^–11^, Zscore = -6.678) in females and (*P* = 3.29 × 10^–12^,Zscore = -6.965) in males. The general meta-analyzed results are summarized with Manhattan plot (Fig. 3A) and the genomic regions around each of the genome-wide significant pQTL variants are represented with regional association plots (Fig. 3B). The trans-pQTL SNP rs12634152 on chromosome 3 which significantly correlated with IL-12B mapped to the *LPP* (Lipoma-preferred partner) locus. Interestingly, genetic polymorphisms in the *LPP* gene are potential risk factors for autoimmune diseases such as celiac disease and Addison’s diseases [23, 24]. Table 2 shows the lead pQTL results for all the 66 proteins interrogated in the females. Suggestive associations for each cohort identified in the females after pQTL mapping and meta-analysis are presented in Table S2.

**Table 2:** Summary statistics of index-pQTLs for females after meta-analysis

MarkerName	Allele1	Allele2	Weight	Zscore	P.value	Direction	chr	pos	Protein
**rs1985329**	**t**	**c**	**359**	**-10.689**	**1.15E-26**	**–**	**2**	**103058208**	**IL.18R1**
**rs4939488**	**t**	**c**	**359**	**7.983**	**1.43E-15**	** + + **	**11**	**60786474**	**CD6**
**rs1204494**	**t**	**c**	**359**	**-7.017**	**2.27E-12**	**–**	**19**	**8060420**	**CCL25**
**rs2843699**	**t**	**c**	**359**	**-6.678**	**2.43E-11**	**–**	**21**	**34663850**	**IL.10RB**
**rs2041190**	**a**	**g**	**359**	**6.336**	**2.36E-10**	** + + **	**17**	**32634541**	**MCP.2**
**rs608574**	**c**	**g**	**359**	**-6.167**	**6.95E-10**	**–**	**2**	**31833066**	**IL18**
**rs12634152**	**t**	**c**	**359**	**-6.116**	**9.58E-10**	**–**	**3**	**188121019**	**IL.12B**
**rs67015567**	**t**	**c**	**359**	**-5.7**	**1.20E-08**	**–**	**3**	**32828703**	**CX3CL1**
**rs12977062**	**t**	**c**	**359**	**5.476**	**4.34E-08**	** + + **	**19**	**54755922**	**PD.L1**
rs175133	t	c	359	-5.414	6.17E-08	–	11	60859791	CCL4
rs62094708	a	g	359	-5.397	6.78E-08	–	18	56159522	Flt3L
rs6486739	a	g	359	5.391	7.01E-08	+ +	12	129440318	CST5
rs13075467	t	c	359	-5.338	9.39E-08	–	3	135609551	CXCL6
rs1954112	t	c	359	-5.304	1.13E-07	–	14	86674145	CD244
rs10816341	t	c	359	-5.293	1.21E-07	–	9	98440825	CASP.8
rs1880614	t	c	359	5.255	1.48E-07	+ +	7	53247740	TRANCE
rs1741298	t	c	359	-5.25	1.52E-07	–	20	4133990	HGF
rs1959748	t	c	359	5.248	1.53E-07	+ +	14	86683256	CCL3
rs7710132	c	g	359	5.244	1.57E-07	+ +	5	51345337	TGF.alpha
rs56875031	c	g	359	-5.235	1.65E-07	–	8	6252045	FGF.21
rs6842405	t	c	359	5.211	1.88E-07	+ +	4	179558777	IL10
rs36018306	t	g	359	-5.195	2.04E-07	–	19	52655428	OSM
rs4299800	c	g	359	-5.18	2.22E-07	–	6	169337246	FGF.23
rs33234	a	g	359	5.179	2.24E-07	+ +	12	31030018	EN.RAGE
rs562966	t	c	359	5.168	2.36E-07	+ +	11	120076116	TNFRSF14
rs16922761	t	c	359	5.162	2.44E-07	+ +	11	95708152	MMP.10
rs2191835	t	c	359	-5.148	2.63E-07	–	2	229942970	IL.17A
rs6460958	a	g	359	-5.13	2.89E-07	–	7	12572904	CXCL9
rs7936105	t	c	359	-5.03	4.91E-07	–	11	77610834	TNFB
rs10270821	t	c	359	-5.02	5.18E-07	–	7	124051449	TNFRSF9
rs9270845	t	c	359	-4.999	5.77E-07	–	6	32570571	MCP.1
rs7818631	t	c	359	4.976	6.48E-07	+ +	8	96427828	FGF.19
rs2102759	a	g	359	-4.963	6.93E-07	–	2	161866727	CCL23
rs13254474	t	c	359	-4.959	7.08E-07	–	8	2918923	IL8
rs7843880	a	g	359	-4.944	7.67E-07	–	8	9099173	CCL11
rs73085912	a	g	359	4.92	8.67E-07	+ +	3	54197988	MMP.1
rs17523444	a	g	359	-4.919	8.69E-07	–	5	15657540	VEGFA
rs324347	a	g	359	4.91	9.09E-07	+ +	5	104092939	OPG
rs3772382	t	c	359	4.908	9.19E-07	+ +	3	24185307	LIF.R
rs2303147	t	c	359	-4.906	9.28E-07	–	19	49143025	CD5
rs396832	a	g	359	4.903	9.45E-07	+ +	21	37299882	IL7
rs35617738	a	g	359	-4.894	9.87E-07	–	3	171187483	CCL28
rs1943821	t	c	359	4.891	1.00E-06	+ +	18	70986060	uPA
rs9430101	t	c	359	4.863	1.15E-06	+ +	1	213014295	CCL19
rs8033014	a	g	359	4.863	1.16E-06	+ +	15	50437063	X4E.BP1
rs4076388	t	c	359	4.85	1.23E-06	+ +	3	72333947	ST1A1
rs12403928	t	c	359	4.838	1.31E-06	+ +	1	190564863	CDCP1
rs10733202	t	g	359	-4.838	1.31E-06	–	9	10027632	CXCL10
rs10832590	t	c	359	-4.815	1.47E-06	–	11	16318856	CD40
rs1342420	t	c	359	4.814	1.48E-06	+ +	20	6138415	TWEAK
rs980325	a	g	359	-4.814	1.48E-06	–	6	153148350	NT.3
rs8105276	a	g	359	4.8	1.59E-06	+ +	19	9101857	CCL20
rs654186	a	t	359	4.797	1.61E-06	+ +	6	132640349	TRAIL
rs16887551	a	g	359	-4.796	1.62E-06	–	8	38470624	AXIN1
rs62273562	a	g	359	4.758	1.95E-06	+ +	3	102908341	MCP.4
rs2266374	t	c	359	-4.746	2.08E-06	–	10	14736230	ADA
rs10916078	a	g	359	4.743	2.10E-06	+ +	1	223857726	SIRT2
rs2368328	a	g	359	-4.722	2.33E-06	–	10	28654250	CXCL11
rs4547370	t	g	359	-4.71	2.48E-06	–	17	63281454	CSF.1
rs75956752	t	c	359	-4.695	2.67E-06	–	13	90524286	CD8A
rs73159896	t	g	359	4.685	2.80E-06	+ +	22	17295981	CXCL1
rs55984748	t	g	359	-4.672	2.98E-06	–	20	739264	IL6
rs2092212	c	g	359	-4.645	3.40E-06	–	22	43318948	SCF
rs6727186	t	c	359	-4.612	3.98E-06	–	2	69103817	DNER
rs9267393	a	c	359	-4.585	4.55E-06	–	6	31469365	LAP.TGF.beta.1
rs9324265	a	g	359	-4.566	4.98E-06	–	13	112835484	CXCL5

### Colocalization analysis implicates sex-specific pQTL loci

**Fig. 4:**
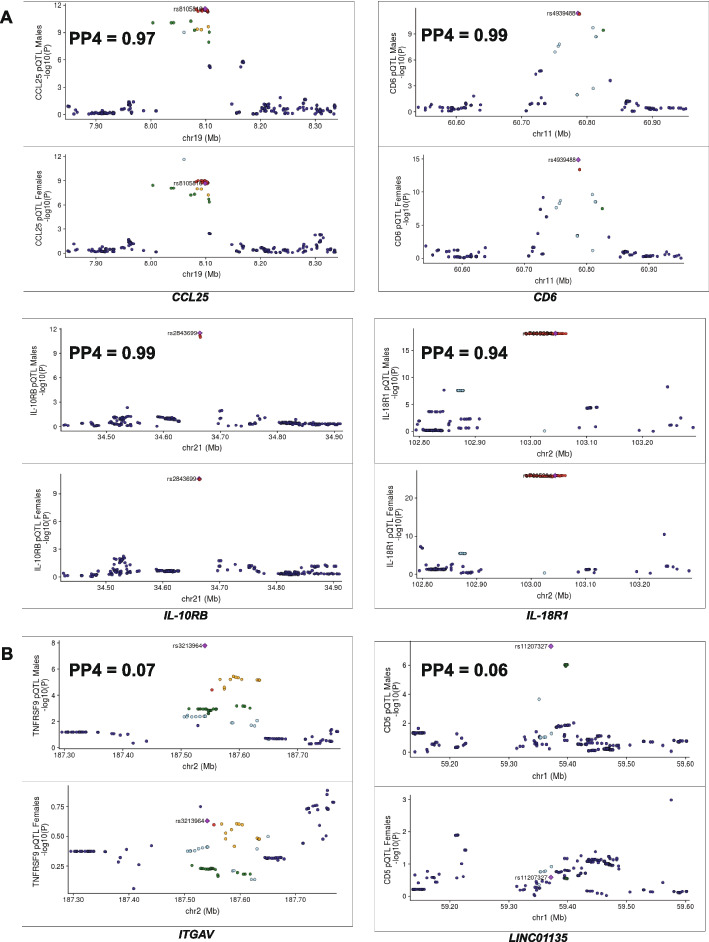
Illustration of colocalization analysis results in males. **A** Locus comparison plots of shared genomic loci between males and females (**B**) Locus comparison plots of male-specific genomic loci. The protein names and -log_10_ association *p*-values are displayed on the vertical axis and the names of the loci and chromosomes are represented on the horizontal axis. The posterior probability values (PP4) are also indicated on the plot

Next, to identify genomic regions that are shared between males and females or unique pQTLs variants, we performed genetic colocalization analysis for all the genome-wide significant loci. A tested region with posterior probability (PP4 >  = 0.75) show a common association in both males and females. In the males, we compared the six significant loci with the same regions in the females. There was strong evidence of shared causal variant with PP4 values ranging from 0.94 to 0.99 in four genomic loci: *CCL25, CD6, IL10RB and IL18R1* (Fig. 4A.). Interestingly, at the *LINC01135* and *ITGAV* loci (Fig. 4B), the PP4 values were just 0.06 and 0.07 respectively, which suggests lack of shared causal variant between sexes. In fact, index SNP look-up showed that the index SNPs in these regions specifically regulate proteins in males. For example, at the *LINC01135* locus, the upstream variant rs11207327 showed strong association with CD5 (*P* = 4.85 × 10^–8^, Zcore = -5.457) in males but was not significant in females (*P* = 0.26, Zscore = -1.126). We also observed a higher PPH1 value (0.693) and a lower PP3 value of 0.0185, supporting the evidence that only males have significant associations in the tested region (Table S3). Individuals carrying the G allele on average produced the highest abundance of CD5 levels. Similarly, at the *ITGAV* locus, while the index SNP rs3213964 surpassed the genome-wide significant threshold (*P* = 1.63 × 10^–8^, Zscore = -5.647) in the males, this effect was completely masked in the females (*P* = 0.23, Zscore = -1.188). This observation was further by supported by the higher PP1 and lower PP3 values 0.894 and 0.0069 (Table S3).

We observed on average higher production of TNFRSF9 levels for individuals with the A allele. To elucidate whether the identified two male-specific loci were not driven by sample size variation or up-scaling of sample size via meta-analysis, we interrogated such effect in the biggest cohort (500FG) with comparatively similar sample size (Males = 184, females = 187) and a similar pattern was observed (Figure S1A). Of note, of all the male-specific loci detected, the lead pQTL SNPs were all trans-pQTLs.

**Fig. 5:**
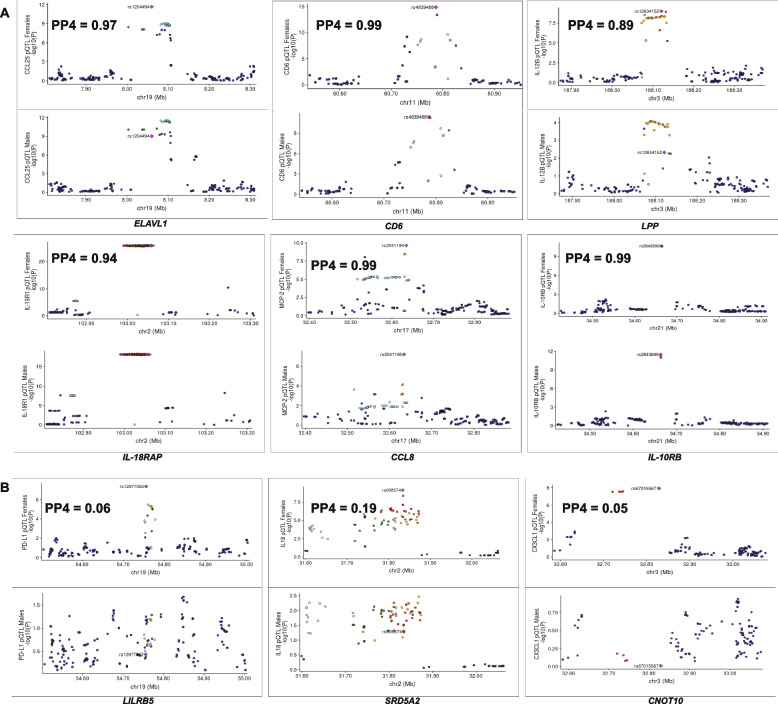
Illustration of colocalization analysis results in females. **A** Locus comparison plots of shared genomic loci between males and females (**B**) Locus comparison plots of female-specific genomic loci. The protein names and strength of association (-log_10_ *p*-values) are displayed on the vertical axis against the chromosomal physical position on the horizontal axis. The posterior probability values (PP4) are also indicated on the plot

We next performed similar colocalization analysis in the nine loci detected after pQTL mapping in females. For six loci, namely *IL-10RB, CCL8, IL-18RAP, LPP, CD6, and ELAVL1* we did not observe any female-specific effects as affirmed by the strong colocalization with PP4 values ranging from 0.89 to 0.99 (Fig. 5A). On the other hand, no evidence of shared causal variant was detected in three loci and the lead SNPs in these regions exhibited trans-association only in females (Fig. 5B). For example, at the *CNOT10* locus (PP4 = 0.05), the downstream SNP rs67015567 on chromosome 3, linked with CX3CL1 levels was significant in females (*P* = 1.2 × 10^–8^, Zscore = -5.70) but not in the males (*P* = 0.98, Zscore = -0.028). While the females carrying the C allele on averaged produced the highest level of CX3CL1, we observed the contrary in the males. Also, at the *LILRB5* locus (PP4 = 0.06), while the association of SNP rs12977062 on chromosome 19 with PD-L1 reached genome-wide significant in females (*P* = 4.34 × 10^–8^, Zscore = 5.476), no significant association was detected in males (*P* = 0.37, Zscore = -0.901). Similarly for PD-L1 levels, females with the T allele produced the highest levels while their counterpart males produced the least. We further observed female-specific effect at the *SRD5A2* locus (PP4 = 0.19) where the association strength of the lead SNP rs608574 on chromosome 2 with IL18 was significant (*P* = 6.95 × 10^–10^, Zscore = -6.167) in females, but not in males (*P* = 0.051, Zscore = -1.952). Individuals carrying the C allele produced the highest levels of IL18 but the average levels for the C and G alleles were comparable in the males. The female-specific genetic variants were also apparent in the analysis considering only the largest cohort (Figure S1B), suggesting that sample size differences cannot account for this effect. In all the three genomic regions, we observed lower PP2 values compared to PP1 values, suggesting the presence of significant associations only in. females (Table S4). Furthermore, we observed an interaction effect between sex and the genotypes of the sex-specific pQTLs, and all but one was statistically significant (Figure S2, Table S5), supporting the robustness of the findings.

#### Conditional analysis identifies secondary pQTL signals

Colocalization analysis with coloc is built under the assumption of a single causal variant per trait and we observed instances of complex LD relationship between some index pQTL variants and neighboring SNPs. For example, variants associated with TNFRSF9 (Fig. 2) and IL18 and PDL1 (Fig. 3) among the males- and females- specific loci. After performing conditional analysis on the top pQTL variants, as summarized in Table S6, no genetic variants reached the significant threshold (pC < 5 × 10^–8^) for IL18 and CX3CL1 proteins. In the case of TNFRSF9 conditioned on rs3213964, 28 pQTL variants were significant, However, these variants showed various degree of LD correlation with the top pQTL variant (R^2^ ranging from 0.239 to 0.754). Interestingly, for CD5 conditioned on rs11207327, the four significant pQTL variants (rs2764912, rs11207331, rs10889121, and rs4912382) have no correlation with the top SNP (R^2^ ranging from 0 to 0.1). Similar observation was made for PDL1 conditioned on rs12977062. The four significant pQTL variants (rs2361797, rs73058787, rs380731, and rs36068997), correlated poorly with the index pQTL (R^2^ ranging from 0 to 0.1), suggesting the presence of multiple or independent associations at the sex-specific loci.

### Exploring the proportion of sex-specific pQTLs across the genome

**Fig. 6:**
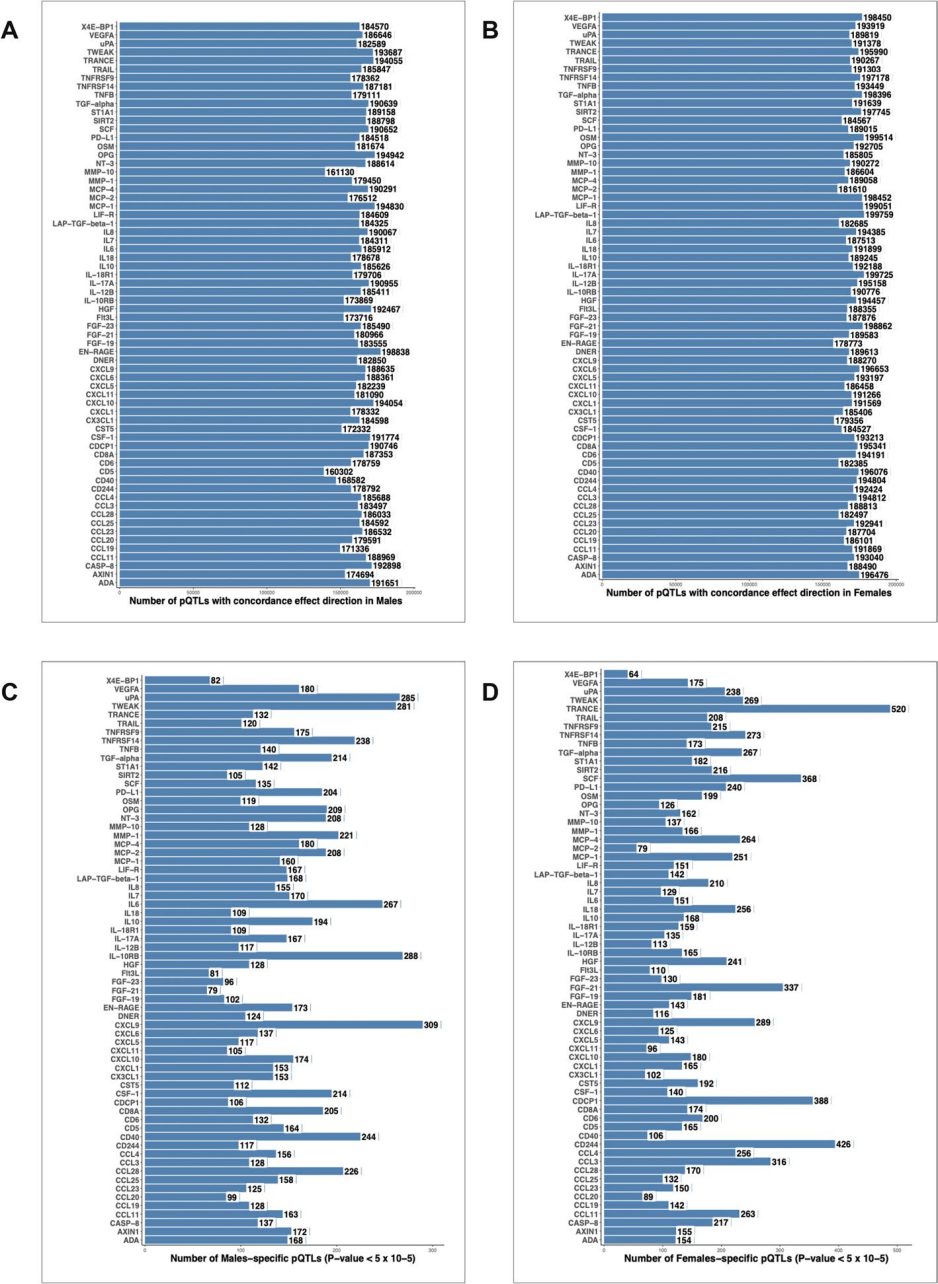
Distribution of pQTLs after meta-analysis in both cohorts. Total number of pQTL variants with consistent effect size direction in both cohorts after meta-analysis (*P* < 0.05) in males (**A**) and in females (**B**). **C** Number of male-specific pQTL variants with strong suggestive cut-off (*P* < 5 X 10^–5^) per protein after meta-analysis of both cohorts in males. **D** Number of female-specific pQTL variants with strong suggestive cut-off (*P* < 5 X 10^–5^) per protein after meta-analysis of both cohorts in females

After identifying sex-specific pQTLs using the genome-wide significant threshold, we next wondered to what extent the pQTLs are sex-dependent if we reduce the statistical threshold. To do this, we selected pQTL variants for each of the 66 proteins used for meta-analysis separately for each sex at a nominal threshold (*P* < 0.05). In the males, the total number of pQTL variants association (*P* < 0.05) range between 160,302 and 198, 838 depending on the protein we tested (Fig. 6A). The number of these pQTLs with significant association (*P* < 0.05) only in males ranged between 152,955 and 189,844 (Figure S3A), which in percentage terms, spans between 3.80% to 4.71% of the total genetic variants of over 4.0 million interrogated (Figure S3C). In females, the identified pQTLs (*P* < 0.05) with consistent effect size direction in both cohorts for all the 66 proteins ranged from 178,773 to 199,759 (Fig. 6B). The number of these pQTL variants that were significant (*P* < 0.05) only in females ranged from 169,779 to 191,123 (Fig. 6B) which shows a percentage of 4.21% to 4.74% (Figure S3C).

Aiming to minimize the degree of false positive associations, we next applied a strong suggestive association threshold (*P* < 5 X 10^–5^). The numbers of sex-specific pQTLs ranged from 79 to 309 for the males (Fig. 6C) and from 64 to 520 (Fig. 6D) in females. The limited percentage of sex-specific pQTLs at a lenient threshold or the limited numbers of sex-specfifc pQTL variants (*P* < 5 X 10^–5^) relative to all the SNPs tested, suggests that most of the genetic variants associated with protein abundances act in sex independent manner. This is supported by the findings of colocalization analysis among the genome-wide significant hits, whereby approximately 67% (males = 4/6, females = 6/9) of pQTLs have similar effects in both sexes.

### Functional and regulatory annotation of sex-specific pQTLs

**Fig. 7:**
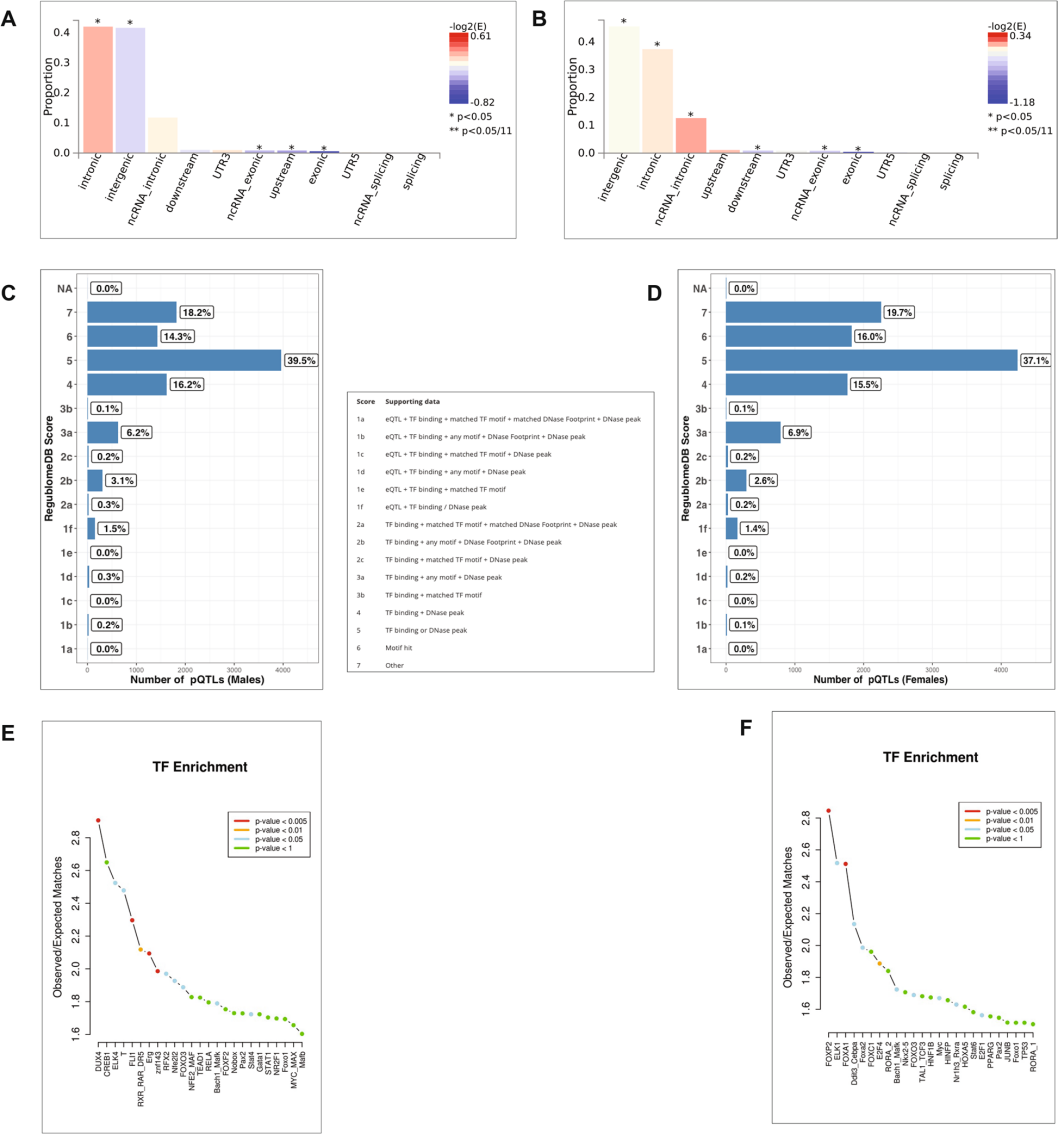
Illustration of functional annotation results of sex-specific pQTLs (*p* *value* = *5* × *10*^*–5*^). Distribution of sex-specific pQTL variants’ functional consequences in males (**A**) and females (**B**). Bar plots distributions of RegulomeDB scores indicating the regulatory potential of pQTL variants for males (**C**) and females (**D**). Interpretation of the scores is sandwiched in between the bar plots. Line plots of Transcription factor enrichment analysis of male-specific pQTL variants (**E**) and female-specific pQTL variants (**F**). The top 25 enriched TFs are ploted on the x-axis and the level of significance are indicated in the color legend

The functional consequences of the sex-specific pQTLs with suggestive evidence of association (*P* < 5 × 10^–5^) shows that most of these pQTLs are within introns and intergenic regions, for both the males (Fig. 7A) and females (Fig. 7B). The sex-specific pQTLs were least represent in coding genomic regions such as UTRs and exons. We next examined the regulatory potential of the of the sex-specific pQTLs and observed that 39.5% and 37.1% of the pQTLs identified had RegulomeDB score of 5 for both the males (Fig. 7C) and females (Fig. 7D) respectively. We observed significant enrichment of pQTLs with RegulomeDB score of 5 among genetic variants with RegulomeDB scores in the database based on chi-squared test (X-squared = 754.57, df = 1, *p*-value = 2.26 × 10^–16^). This observation indicates that most of the identified pQTLs are likely to alter transcription factor binding sites (TFBs) and are therefore regulatory. In fact, lower RegulomeDB scores provide evidence that, the pQTL variants are located in a functional region and pQTL variants falling within the category one was as low as 1.5% and 1.4% in the males and females respectively.

Given that most of the identified pQTLs were predicted to alter TF binding sites, we mapped the sex-specific pQTL variants with suggestive evidence association to TFBs to uncover the affected transcription factors (TFs). Several TFs were identified after TF enrichment analysis in the males (Fig. 7E) with DUX4, CREB1 and ELK4 being the three most enriched TFs. In the females (Fig. 7F), TFs such as FOXP2, ELK1, and FOXA1 were mostly enriched. Knowing that pQTL variants change or disrupts TF-binding is crucial to understand the molecular mechanisms of how pQTL variants impact protein abundances.

### Biological interpretation of TFs and gene sets

**Fig. 8:**
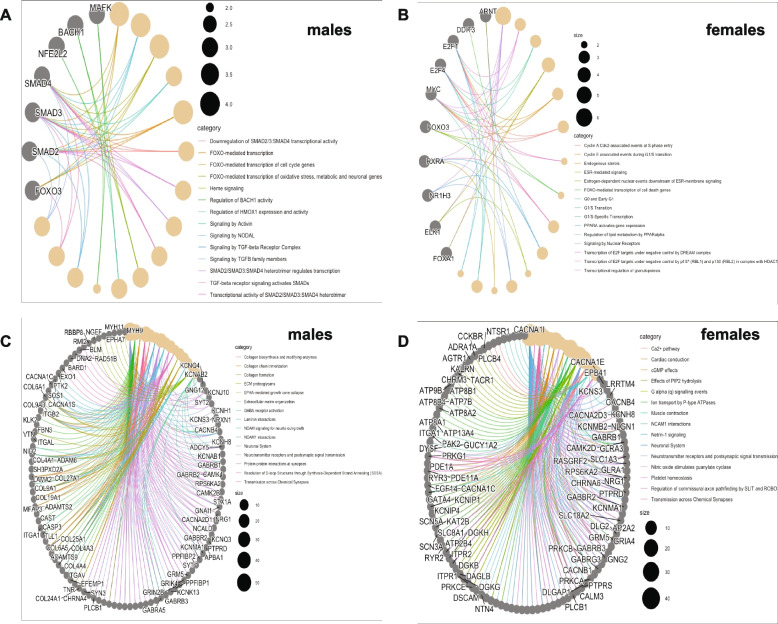
Biological interpretation of sex-biased pQTLs variants (*p* *value* = *5* × *10*^*–5*^). Pathway enrichment analysis using significantly enriched TFs matched to the sex-specific pQTL variants in males (**A**) and in females (**B**). Pathway enrichment analysis of annotated gene sets in males (**C**) and in females (**D**). *P*-value < 0.05 with Bonferroni multiple correction method was set for significantly enriched terms (category). Genes and or TF names related to the pathways are displayed on the circular plot

Next, we sought to gain mechanistic insight into the predicted TFs (see methods) and curated gene sets (mapping pQTL variants to genes) from the sex-specific pQTL variants (*P* < 5 × 10^–5^). To do this, we performed over-representation analysis to identify enriched biological pathways. Among the predicted TFs (Table S7 & Table S8), we selected significant TFs with *P*-value < 0.05 which means that only TFs identified not by chance are used for further analysis. According to the Reactome database, several pathways were significantly enriched for the TFs in the males (Fig. 8A, Table S9). Pathways such as FOXO-mediated transcription of cell cycle genes and Signaling by Activin and Signaling by NODAL were identified. We also identified enrichment of TFs in pathways such as Endogenous sterols, ESR-mediated signaling and Estrogen-dependent nuclear events downstream of ESR-membrane signaling in the females (Fig. 8B, Table S10). GO ontology analysis shows that the TFs for the males (Figure S4A) and females (Figure S4B) are generally involved in metabolic, biological and developmental processes.

To explore biological relevance of the gene sets using the Reactome database, while pathways such as Extracellular matrix organization, Collagen formation, Collagen biosynthesis and modifying enzymes and Protein–protein interactions at synapses were identified among the top 15, others such as Regulation of insulin secretion were significant pathways discovered for males (Fig. 8C, Table S11), while for females the mapped gene-sets were enriched for pathways such as Ca2 + pathway, Platelet homeostasis, Muscle contraction (Fig. 8D, Table S12). The Neuronal system however appeared as common pathway in both sexes. Gene ontology analysis for males (Figure S5A) and females (Figure S5B) revealed that the curated genes-sets are overall involved in biological, metabolic, developmental and reproduction processes. The identification and understanding of sex-specific pathways is crucial to design effective therapeutics, especially for the diseases that are expressed differently between sexes.

### Sex-dependent pQTL variants overlap with sex-related molecular traits

We cross referenced the genome-wide significant index pQTL variants with other phenotypic traits, not limited to gene expression, metabolites, and epigenetic markers, to explore any potential biological pleiotropy. Overall, in the males, all the pQTLs variants showed association with various diseases or traits and expression levels of genes (Figure S6). For example, male-specific pQTL rs3213946 is also eQTL for several genes (*ITGAV, FAM171B, ATP6V1B2 and ZC3H15*) and associated with diseases such as inflammatory bowel disease, coronary artery disease, and attention deficit hyperactivity disorder. Another male-specific pQTL rs11207327 linked with CD5 levels also modulates the expression of genes (*JUN, KRT79, TACSTD2*), and it is associated with diseases and traits (Asthma, self-reported haemorrhoids and treatment with estriol product). Evidence of prior associations with metabolites (e.g., X-12441, citrate and Hypoxanthine) and epigenetic markers (e.g., H3K4me1, H3K27ac and cg07810476) were also uncovered.

Similarly, in females, the index pQTL variants are also eQTLs and correlate with other traits (Figure S7). For instance, SNP rs608574 linked with IL18 and showing association only females, regulates genes such as *SRD5A2, CAPN13, SLC30A6, NLRC4, XDH and SPAST,* and it is as well associated to traits and diseases (e.g., height, age started oral contraceptive pill, single delivery by caesarean section, treatment with diltiazem, chronic sinusitis, birth control pills and fistulae involving female genital tract). Another pQTL SNP rs12977062 associated with PD-L1 regulates multiple genes, not limited to *GP6, TPM3P6 and LILRB5*. Associations with epigenetic markers (e.g., percent-splice-in and cg15691140), other traits and diseases (treatment with noriday tablet, convulsions, birth weight, height, treatment with tridestra tablet and atherosclerosis) were also detected. These observations cross validate the identified pQTL variants and justifies the roles of pQTLs in explaining the mechanisms of diseases.

## Discussion

This study investigated the contribution of host genetics to sex differences in inflammatory proteins production capacity by conducting meta-analysis of pQTL summary statistics in males and females separately using two population-based cohorts. Given the well-known sexual dimorphism in most complex traits and diseases, there is growing awareness for large scale genetic studies to unravel sex-specific genetic factors.

In this study, we provide evidence for the contribution of common autosomal SNPs for differences in inflammatory biomarker between sexes by identifying genome-wide significant pQTLs in the sex-stratified analyses for males (6 pQTLs) and for females (9 pQTLs). In general, we observed that majority of the pQTL effects, from the tested autosomal SNPs (4,028,465) on the 66 proteins, are shared between males and females with approximately 5% displaying sex-specific effects. This observation is concordant with previously published evidence, highlighting the lack of strong sex-biased genetic effects on complex traits. For example, large scale twin studies across 50 human traits observed sex-specific genetic factors for 25% of the traits with limited sex-specific genetic variants except for the apparent puberty-related traits [25]. Another study targeting specifically 20 neuropsychiatric and behavioral traits, showed that between-trait genetic correlation estimates were not significantly different between males and females [26]. In fact, a recent study showed that sex-specific eQTLs do not account for the sex-specific trait associations and demonstrated through power analysis that millions of GWAS samples are required to detect sex-specific trait associations driven by sex-biased eQTLs [27]. Therefore, even though the limited percentage of sex-specific pQTLs identified in our study is in line with previous findings, statistical power could account for the minimal percentage observed as well.

Albeit most of the genome-wide pQTLs appeared to exhibit shared effect between males and females, colocalization analyses also highlights specific genomic-loci with sex-biased effects. The two loci harboring male-specific pQTLs are *LINC01135* and *ITGAV*, which are lncRNA and protein coding genes respectively. While the function of *LINC0113* gene is not completely known, recent study has demonstrated its role in prostate cancer- skin cancer affecting males [28]. Interestingly, the sentinel pQTL variants at these loci (*ITGAV* (rs3213964) and *LINC0113* (rs11207327)) were previously reported to be associated with various diseases and intermediate traits such as gene expression and metabolites which are likely to mediate the observed associations. The SNP rs3213964 at *ITGAV* gene is evident through cross validation approach to be associated with male-related phenotypic traits such as malignant neoplasm of testis, sitting height and self-reported testicular cancer which supports the stronger associations detected in males.

Furthermore, three pQTLs (*CNOT10* (rs67015567), *LILRB5* (rs12977062) and *SRD5A2* (rs608574)) were identified to modulate proteins specifically in females. The *CNOT10* gene is related to metabolic pathways as it is predicted to be involved in catabolic process and also participates in negative regulation of translations. The *LILRB5* gene is involved in the regulation of immune pathways and its role in negative regulation of cytokine production is reported [29]. We further showed that the lead SNP rs12977062 at the *LILRB5* gene is correlated with expression of other genes and epigenetic markers. The *SRD5A2* gene is involved in pathways such as metabolism of steroid hormones and widely known for the regulation of testosterone biosynthetic process. Although testosterone is traditionally thought of as males sex hormones, females also produce it but in lower quantities [30]. Supporting this, the *SRD5A2* gene also converts progesterone or corticosterone into their corresponding 5-alpha-3-oxosteroids [31] and mutations in the *SRD5A2* gene has been associated with human intersex condition termed as pseudohermaphroditism [32]. These unique characteristics of this gene makes it possible to function in either sex, but we speculate that genetic polymorphisms in this gene might regulate phenotypes differently. In line with this claim, the lead SNP rs608574 at *SRD5A2* gene overlap with female dominated traits, among others treatment with livial tablet and fistulae involving female genital tract, supporting the stronger association of this variants specifically in females. Surprisingly, all the genome-wide significant pQTL variants identified with sex-specific effects are trans-pQTLs, which suggests that the uncovered pQTL variants may not have direct effect on the protein coding gene but other proteins and pathways are involved in the mechanism underlying the genetic sex-differences in protein levels.

Interestingly and supporting this argument, over-representation analysis of genes mapping to the female-specific pQTLs implicated pathways such as Ca2^+^ pathway and cardiac conduction which has functional interplay in cardiovascular diseases, with increased susceptibility among females in developed countries [33]. Regulation of calcium ion (Ca2^+^) cycle is important for cardiac contraction and relaxation and estrogen levels in plasma can affect cardiac function [34]. Aside geometric differences of the healthy heart, functional differences such as contractility exist between sexes with smaller cardiac output and larger ejection fraction are reported in females compared to males [35]. On the other hand, pathway analysis highlighted the function of genes annotated to the male-specific pQTLs to collagen formation and protein–protein interaction.

Furthermore, large proportion of these pQTLs variants are TFBS variants, suggesting their gene regulatory role. The TFs identified are enriched among sex-related pathways. For instance, in the females are Endogenous sterols, ESR-mediated signaling and Estrogen-dependent nuclear events downstream of ESR-membrane signaling. Higher concentrations of endogenous sex steroids and mutations of estrogen signaling receptor (ESR) genes are some of the underlying causes of breast cancer [36, 37]. In the males, pathways such as signaling by activin and signaling by nodal were detected. Activin is mainly produced in the male reproductive tract and helps main cell–cell interactions, especially in the testis and prostate [38]. Similarly, the nodal signaling pathway is known to regulate fetal testicular development and uncontrolled nodal signaling has been implicated in testicular cancer [39]. While these downstream observations strongly support the validity of the identified sex-dependent pQTL variants, further mechanistic studies are required to discern the mechanisms by which these pathways drive sex-specific pQTLs.

### Limitations of the study

It is important to acknowledge that, exclusion of the sex chromosome variants is a major limitation. Despite several evidence pointing to the autosomal genomic region to driving phenotypic sex-differences [6], the X chromosome is known to accommodate the largest number of genes related to the immune system [40]. Also, even though, we employed meta-analysis of two different cohorts to increase statistical power, upscaling of the sample sizes and the utilization of methods that consider effect size differences between males and females, could help identify additional genome-wide significant loci. Additionally, increasing the number of proteins by utilizing the Olink explore panel is desirable for future work. Finally, to be able to ascertain whether the identified pQTLs are population-specific or shared, and to subsequently facilitate the translation of the findings into clinical practice, extending this analysis to diverse populations is warranted.

## Conclusion

In conclusion, we identified and characterized sex-specific pQTLs, which is crucial to discerning the underlying mechanisms of complex diseases, traits and biomarkers. Given that proteins determine almost all cellular process, ultimately dictate the phenotypic expression, and dysregulation of proteins are also implicated in several diseases, the identified sex-specific pQTL variants could be use as genetic instruments through mendelian randomization to interrogate how these inflammatory mediators are causally implicated in sex-biased phenotypes and/or diseases. This information will eventually help understand disease biology and also facilitate the development of new therapeutics with strong efficacy in one sex.

## Materials and methods

### Study cohorts

The 500FG cohort is a population-based cohort of healthy individuals of Western European ancestry consisting of 237 males and 296 females with age range of 18-75 years. The 300BCG cohort is another population-based cohort of 325 (142 males and 183 females) healthy individuals of Western European origin with age range of 18–71 years. Sex classification of research participants was based on sex assigned at birth.

Both cohorts are part of the Human Functional Genomics Project (wwww.humanfunctionalgenomics.org), aimed at identifying the genetic and non-genetic determinants of the immune response.

### Measurement of inflammatory protein biomarkers

Inflammatory protein levels were measured using targeted proteomics (Olink® platform). We generated protein abundance of the plasma samples which was quantified using the inflammatory panel of 92 proteins. The Olink data are reported in NPX values (Normalized Protein expression) which are on log 2 scale. Immunoassays utilized by Olink are based on the Proximity Extension Assay (PEA) technology [41], which makes use of oligonucleotide-labelled antibodies binding to their respective protein. When the two antibodies are brought in proximity, a DNA polymerase target sequence is formed, which is subsequently quantified by quantitative real-time polymerase chain reaction (qPCR). Each plate included interplate controls which are used to adjust any potential plate difference. NPX values were intensity normalized with the plate median for each assay as the normalization factor (Intensity Normalization v.2).

### Preprocessing / filtering of protein data and normalization

Samples that did not pass Olink internal quality control or flagged “Warning” were excluded, so as proteins which failed to be quantified in at least 75% of the samples. The remaining 73 and 70 proteins in the 500FG and 300BCG cohorts respectively, with NPX values below the protein-specific detection limit (LOD) were replaced with their corresponding LOD values as recommended by Olink. 66 proteins were common between both cohorts after filtering.

### Genotyping and genetic data quality control

We previously described details of the genotyping, imputation and all quality control procedures for the 500FG [42] and 300BCG [43] cohorts. For the 500FG cohort, DNA samples of approximately 500 individuals were genotyped with the commercially available SNP chip, Illumina HumanOminiExpress-8 v1.0 and DNA samples of 325 individuals were genotyped for the 300BCG cohort using the Infinium Global Screening Array MD version 1.0 from Ilumina SNP chip. The genotype calling for both cohorts were performed using Optical 0.70 with the default settings [44]. Standard pre-imputation quality filters such as excluding variants with call rate ≤ 0.99 Hardy–Weinberg equilibrium (HWE) ≤ 0.0001 and minor allele frequency (MAF) ≤ 0.001. Per sample quality control such as sex discrepancies, cryptic relatedness and population stratification to exclude genetic outliers (17 and 12 in the 500FG and 300BCG cohorts respectively) were applied. Genotyped samples were imputed with the Michigan Imputation server [45], with the Genome of the Netherlands Consortium, (GoNL 2014) and the human reference consortium (HRC r1.1 2016) reference panels for the 500FG and 300BCG cohorts respectively.

We filtered out genetic variants with imputation quality score (R^2^) < 0.3 and MAF cut-off of 5%. A total of 4,296,841 and 4,358,039 SNPs were available for the 300BCG and 500FG cohorts respectively.

### Protein QTL mapping

The association of protein-genotype analysis was performed in a sex-stratified manner for both cohorts separately as protein measurements were not performed at the same time. We used the linear model function in the Matrix-eQTL R package [46] for association analysis. Age of the participants was included in the model which was fitted on the inverse ranked normalized protein concentration. In the 300BCG cohort, a total of 306 (Males = 134 and Females = 172) samples were retained after quality control and with matched genotype–phenotype data. In the case of the 500FG, the total samples used for pQTL mapping was 371 consisting of 184 males and 187 females. In the males, the mean ages are 27.20 and 29.56, and the standard deviations are 12.30 and 14.68 for the 300BCG and 500FG cohorts respectively. In the females also, the mean ages are 24.66 and 24.77 and the standard deviations are 8.60 and 10.98 for the 300BCG and 500FG cohorts respectively. Wilcoxon test did not show any significant age difference between both cohorts in each sex (*P* > 0.05).

### Meta-analysis of males and females pQTL results

The meta-analysis was carried out using fixed effects sample size weighted analysis method implemented in METAL package [47], based on pQTL summary statistics (*p*-values). Here, the analysis which was conducted for males and females from both cohorts separately, was confined to the 66 proteins and 4,028,465 SNPs common between the 500FG and 300BCG cohorts. We extracted SNPs with consistent effect size direction per protein for further analysis.

#### Estimation of study-wide significant threshold

To account for the multiple testing burden, the significant cut-off is determined based on the ratio of the 5% significant level and the product of the number of proteins (66) and the number of tested SNPs (4,028,465). The resulting *p*-value was 1.89 × 10^–10^. Given that few associations surpassed this stringent threshold, we considered the conventional genome-wide significant threshold (5.0 × 10^–8^) together with colocalization analysis to identify key loci exhibiting sex-dependent effect. For sex-specific biological insights, we used associations with strong suggestive threshold of 5.0 × 10^–5^ in one sex which failed to be replicated in the opposite sex at nominally significant threshold of 0.05.

### Colocalization of pQTLs between sexes

Colocalization of pQTLs identified in males and females was performed using Bayesian colocalization method which is implemented in the coloc package in R [48]. The default prior probability and parameters that a random variant in the region is causal to both traits was applied. For each protein-SNP genome-wide significant pair considered for colocalization, SNPs within a window size of 500 kb around the lead SNP were tested. A posterior probability (PP4 >  = 0.75) is considered as strong evidence of colocalization or shared genomic region between the sexes [48]. Other hypothesis tested are PP1 and PP2 which indicates either males or females have significant associations in the tested region and PP3, which indicates that both males and females have significantly unique causal variants. LocusCompareR, being an R package was used for visualizing the results [49].

#### Conditional analysis on index sex-specific pQTL variants

We performed conditional analysis using the GCTA-COJO software [50]. As the software requires effective size and standard error statistic as input, the METAL Zcores from the meta-analysis results were used to estimate the effect size (Beta) and standard error (SE) values using the two equations suggested by previously [51].

1 $${\text{Beta}}={\text{Zscore}}/{\text{sqrt}}\left( {2}^{*}{{\text{MAF}}}^{*}{\left(1-{\text{MAF}}\right)}^{*}\left(\mathrm{ N}+{\text{Zscore}}^2 \right)\right)$$

2 $${\text{SE}}=1/{\text{sqrt}}\left( {2}^{*} {{\text{MAF}}}^{*} {\left( 1-{\text{MAF}}\right)}^{*}\left(\mathrm{ N}+{\text{Zscore}}^2\right)\right)$$

The parameters in the equation are defined as: sample size (N), and minor allele frequency (MAF). The 500FG cohort was used as the LD reference panel. The analysis was confined to regions around 250 kb of the index pQTL variants. A conditional *p*-value (pC) threshold of 5 × 10^–8^ was used to identify secondary hits.

### Functional annotation of sex-specific pQTLs

We selected sex-specific pQTLs with strong suggestive association (5.0 × 10^–5^) for functional annotation. The functional consequences of the pQTL variants on genes functions were explored using the ANNOVAR method in Functional Mapping and Annotation of GWAS (FUMA) [52]. The putative regulatory potential of the pQTLs were also accessed with RegulomeDB [53] which incorporates high-throughput, experimental, different data sources and computational predictions to score genetic variants. RegulomeDB assigns scores ranging from 1 to 6 to help classify SNPs and to determine genetic variants with or with regulatory functionality. Genetic variants with lower scores indicate stronger evidence of residing in a regulatory region.

### Mapping sex-specific pQTLs with transcription factor binding sites

SNP2TFBS web-based annotation tool [54], was applied to study functional effects of genetic variants within transcription factor binding sites (TFBS) of the human genome. Briefly, a SNP’s effect on transcription factor (TF) binding is estimated with position weight matrix (PWM) model for the binding specificity of the corresponding factor. This results in a list of SNPs that overlaps with predicted binding sites of a specific TF. The list of TFs being mapped by the SNPs are generated. The TF enrichment is done by computing the ratio of the observed SNP hits over the expected hits for each TF.

*P*-value less than 0.05 was declared as statistically significant threshold for TF enrichment.

### Pathway over-representation analysis

The SNP2GENE function in FUMA [52] was applied to annotate sex-specific pQTL variants to genes. Default settings in FUMA were used to identify independent lead associations with the following parameters: r^2^ < 0.6 for independent significant SNPs, 2nd r^2^ < 0.1 for index SNPs and a window size of 250 kb to determine LD blocks. The independent lead SNPs are then mapped to genes based on positional and functional information of SNPs. The curated gene sets were used as input for both pathway enrichment analysis and Gene Ontology (GO) slim analysis. Gene Ontology slim analysis provides a higher-level summary or term of GO ontologies or provides a broader overview of the GO ontology terms.

Enrichment of the candidate gene list and TFs were estimated using ClusterProfiler R package for hypergeometric test [55]. Bonferroni multiple testing correction method with q-value < 0.05 was declared as significant pathways. For a broader overview of the annotated terms, GO slim analysis was performed with the WebGsalt tool [56].

### Integration of pQTL variants with other molecular traits: cross validation

We looked up for a range of phenotypes or traits that are associated with the identified genome-wide significant independent pQTLs with Phenoscaner [57], which is a curated database of publicly available summary statistics of large-scale genotype–phenotype associations, not limited to NHGRI-EBI GWAS catalog and dbGAP catalogs. Association results of traits such as gene expression, proteins, metabolites, epigenetics and diseases with our pQTL variants were downloaded and overlap analysis was conducted. A nominal threshold of 1 × 10^–3^ was applied to select SNP-phenotype associations from the database.

### Statistical analyses

All statistical methods and tools used are described under the appropriate sections in the methods. Analysis and visualization were performed in R version 4.10 unless otherwise stated. Two-way Analysis of Variance (ANOVA) was performed to examine the potential interaction between SNP genotypes and Sex. The aov () function in R was used for conducting the analysis and interaction effects were visualized using the interaction. plot () function.

### Supplementary Information

**Additional file 1: Figure S1.** Comparing the genetic effects of sex-specific pQTL in 500FG cohort.** Figure S2.** Visualization of Sex-by-SNP interaction in the 500FG cohort.** Figure S3.** Bar plots distribution of sex-specific pQTLs after meta-analysis.** Figure S4.** Graphical illustration of GO slim results with Transcription Factors (TFs).** Figure S5.** Graphical illustration of GO slim results with curated gene sets.** Figure S6.** Circular barplots summarizing traits associated with male-specific pQTLs.** Figure S7A.** Circular barplots summarizing traits associated with genome-wide significant pQTLs in females.** Figure S7B.** Circular barplots summarizing traits associated with genome-wide significant pQTLs in females.

**Additional file 2: Table S1.** Summary statistics of pQTL mapping and meta-anlysis results in the males group.** Table S2.** Summary statistics of pQTL mapping and meta-anlysis results in the females group.** Table S3.** Summary statistics of genome-wide significant meta-anlysis and colocalization results in the males group.** Table S4.** Summary statistics of genome-wide significant meta-anlysis and colocalization results in the females group** Table S5.** Summary statistics of analysis of variance (ANOVA) results using the 500FG cohort.** Table S6.** Summary statistics of conditional analysis results of sex-specific loci.** Table S7.** List of TFs enriched or linked to male-specific pQTL variants.** Table S8.** List of TFs enriched or linked to female-specific pQTL variants.** Table S9.** Details of enriched pathways of TFs linked with male-specific pQTL variants.** Table S10.** Details of enriched pathways of TFs linked with female-specific pQTL variants.** Table S11.** Enriched pathways of genes mapped to male-specific pQTL variants.** Table S12. **Enriched pathways of genes mapped to female-specific pQTL variants.

## Data Availability

The raw genetic and phenotypic data used to uncover the findings in this study are available from the corresponding author upon reasonable request due to restrictions to avoid compromising research volunteers’ privacy. Data can be acquired using the data request form (http://www.humanfunctionalgenomics.org/site/?page_id=16). No custom code was generated for this study and all software/tools used have been properly referenced at the appropriate sections.
