# Supplementary material for: Sex-biased genetic regulation of inflammatory proteins in the Dutch population
Source: BMC Genomics. 2024 Feb 8;25:154. doi: 10.1186/s12864-024-10065-z (PMC10851559; doi:10.1186/s12864-024-10065-z)
Supplement: Supplementary file 1 — Additional file 1: Figure S1. Comparing the genetic effects of sex-specific pQTL in 500FG cohort. Figure S2. Visualization of Sex-by-SNP interaction in the 500FG cohort. Figure S3. Bar plots distribution of sex-specific pQTLs after meta-analysis. Figure S4. Graphical illustration of GO slim results with Transcription Factors (TFs). Figure S5. Graphical illustration of GO slim results with curated gene sets. Figure S6. Circular barplots summarizing traits associated with male-specific pQTLs. Figure S7A. Circular barplots summarizing traits associated with genome-wide significant pQTLs in females. Figure S7B. Circular barplots summarizing traits associated with genome-wide significant pQTLs in females. [file 12864_2024_10065_MOESM1_ESM.pdf]

Supplementary Information

Figures

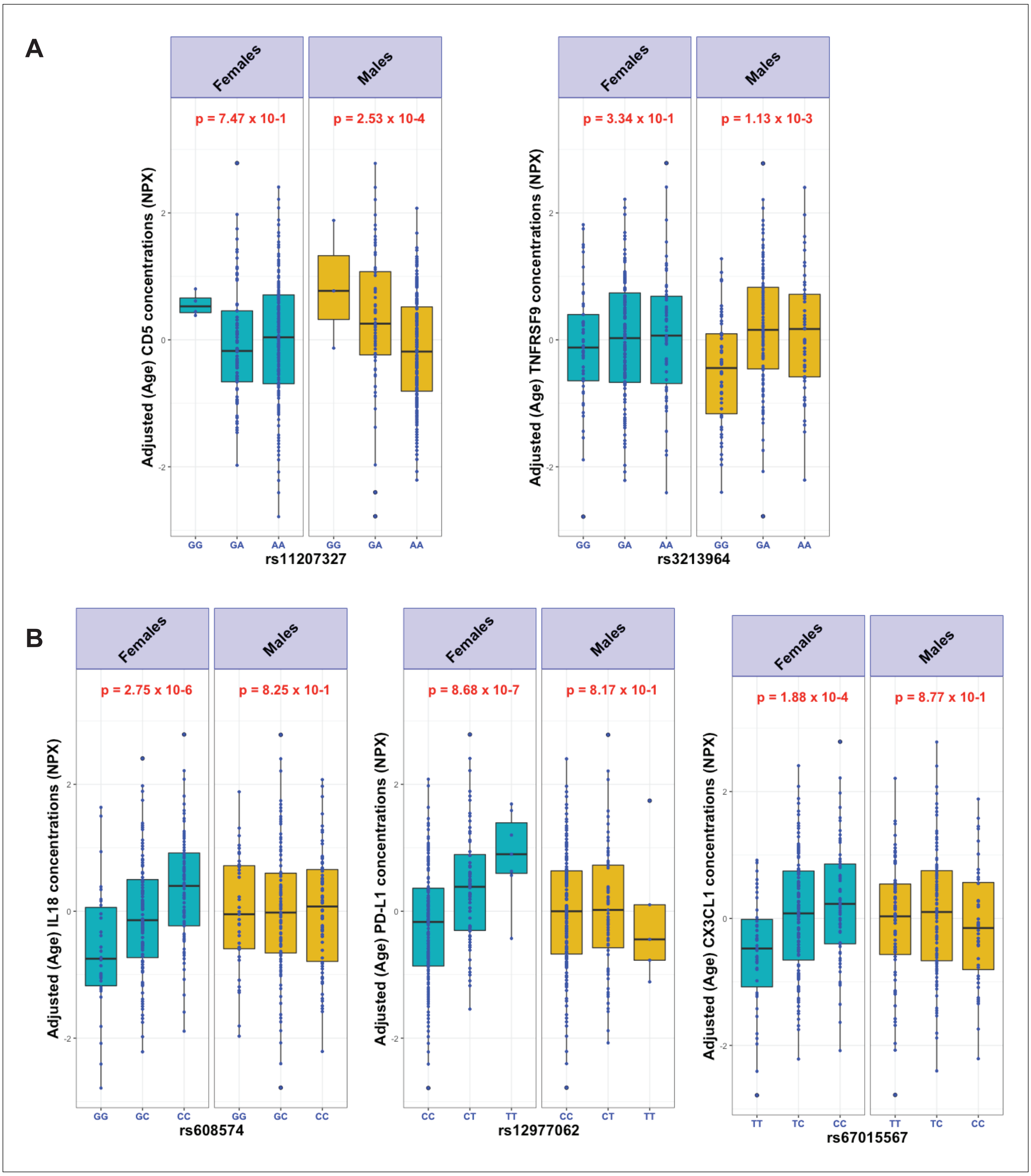

Figure S1: Comparing the genetic effects of sex-specific pQTL in 500FG cohort. *(A) Boxplots of the male-specific pQTL variants stratified by genotypes. (B) Boxplots of the female-specific pQTL variants stratified genotypes. The y axis represents the proteins associated with the SNPs on the x axis. P-values between sexes are displayed on the plots.*

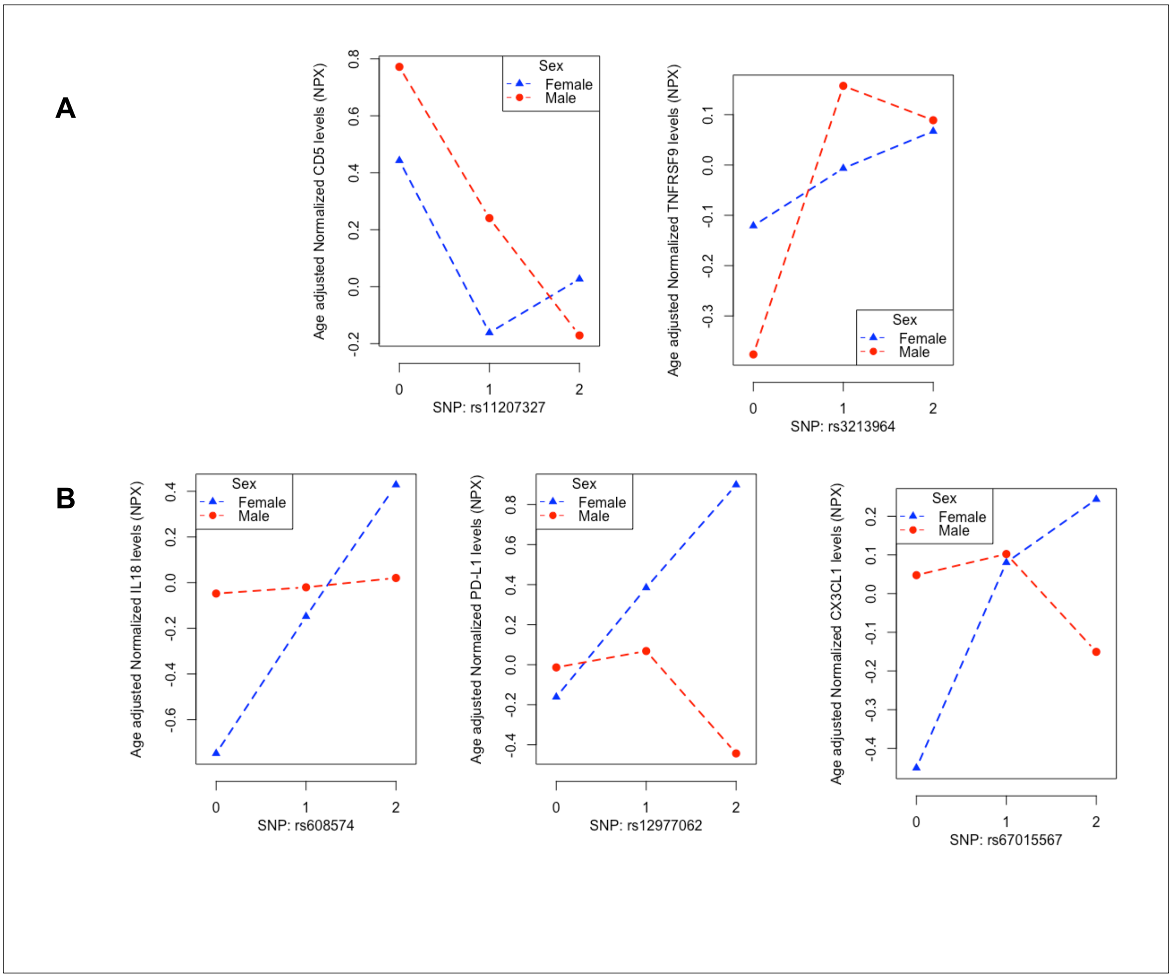

Figure S2: Visualization of Sex-by-SNP interaction in the 500FG cohort.
(A) *Interaction plot of male-specific genome-wide significant pQTL variants (B) Interaction plot of female-specific genome-wide significant pQTL variants. The median values of the proteins are depicted with circle and triangle points for males and females respectively.*

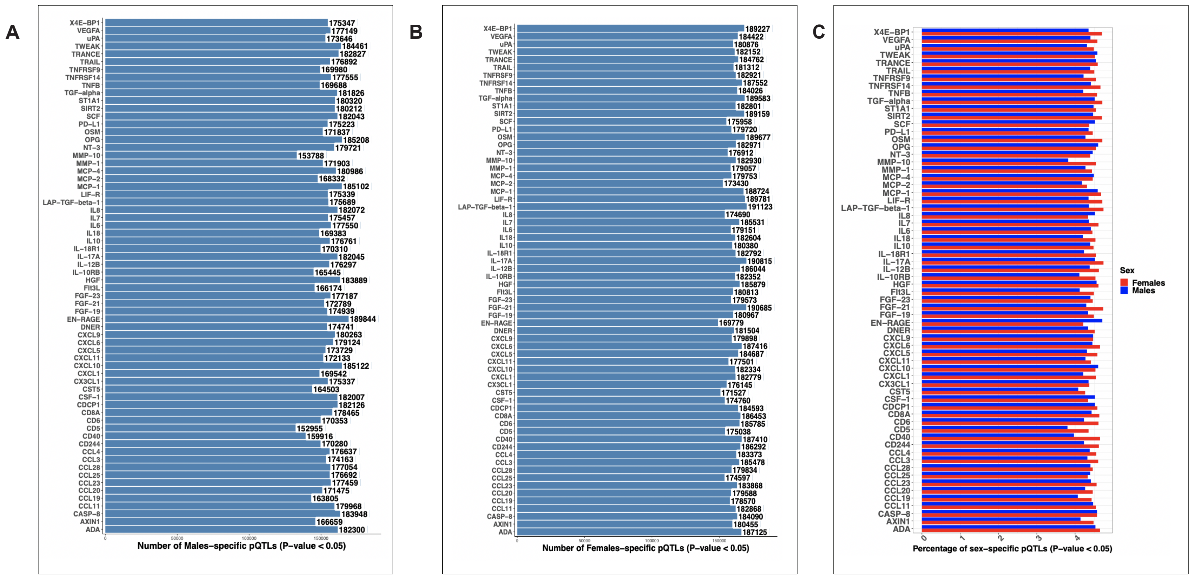

Figure S3. Bar plots distribution of sex-specific pQTLs after meta-analysis.
(A) Number of male-specific pQTL variants per proteins after meta-analysis of both cohorts in males. (B) Number of female-specific pQTL variants per proteins after meta-analysis of both cohorts in females. The length of the bars reflects the number of pQTL variants (C) Percentage of sex-specific pQTL variants estimated based on the numbers displayed in A and B with reference to the total number of tested SNPs (4,028,465). Blue and red bars in the legend represents males and females respectively. The vertical axis shows all the 66 proteins.

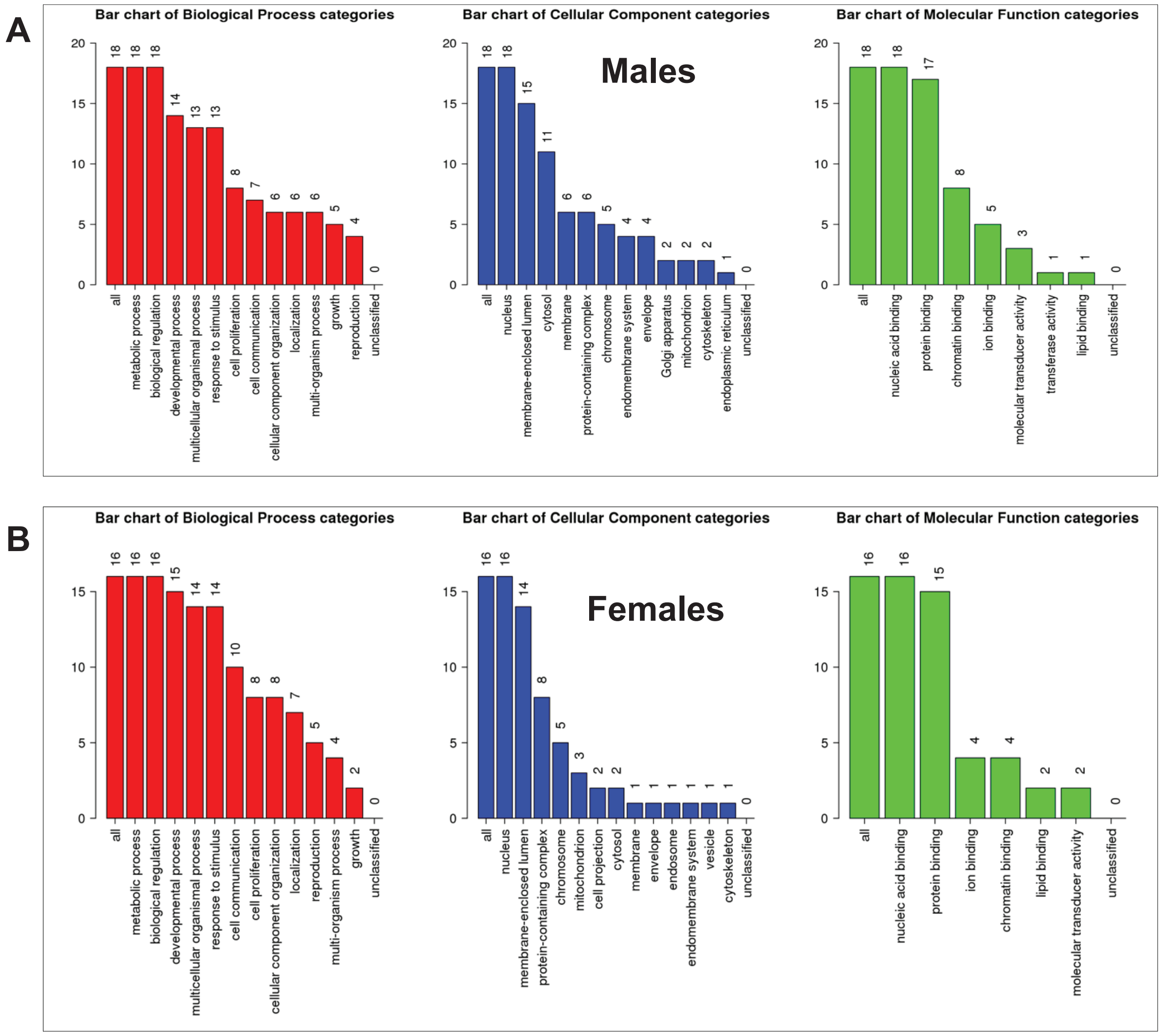

Figure S4: Graphical illustration of GO slim results with Transcription Factors (TFs)
Bar plots depicting the number of TF genes coinciding with the annotated genes in the GO subsets (x axis) which is color coded as red, blue and green for biological, cellular and molecular function processes respectively.

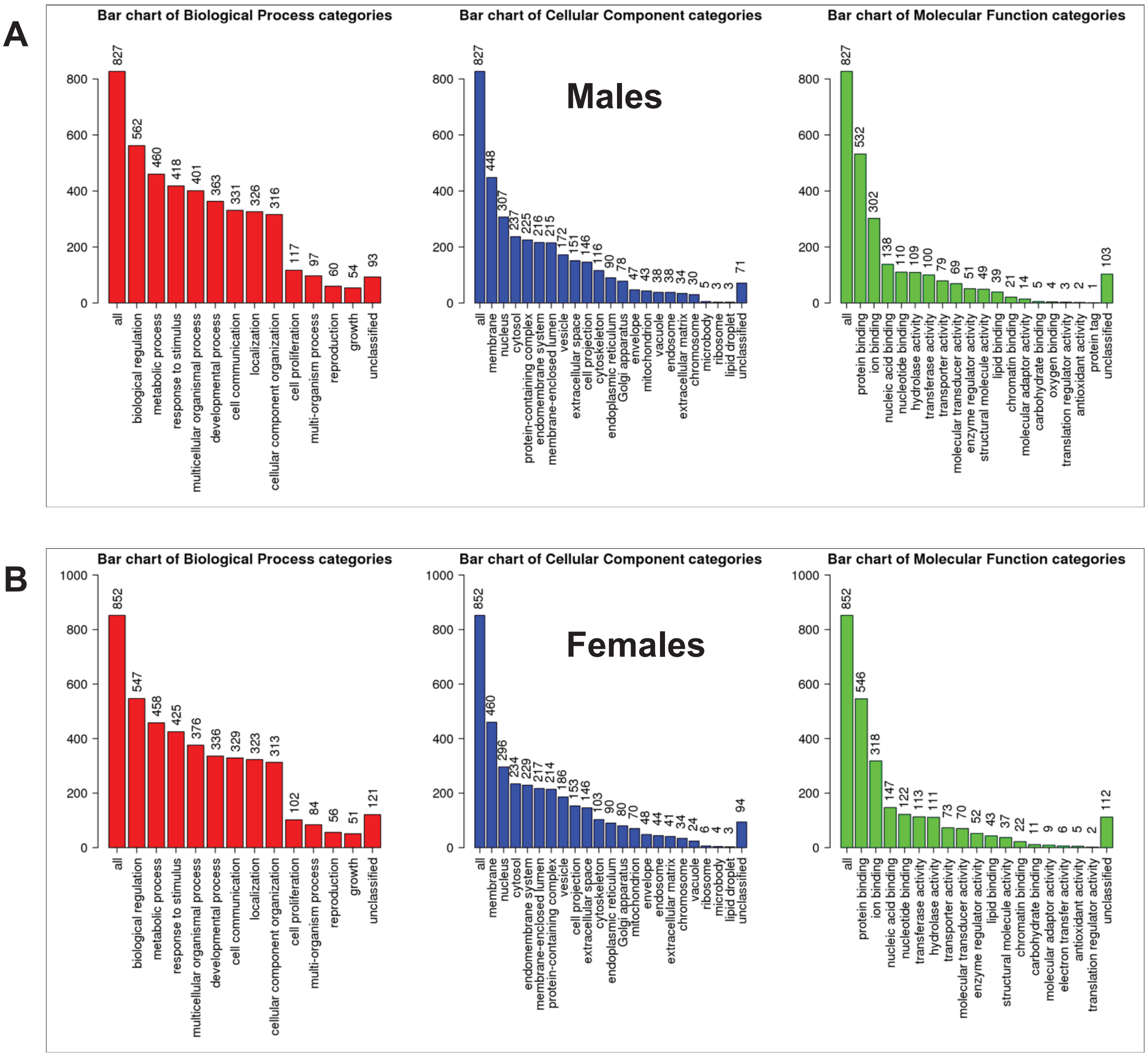

Figure S5: Graphical illustration of GO slim results with curated gene sets
Bar plots depicting the number of genes mapping to sex-specific pQTL varaints, coinciding with the annotated genes in the GO subsets (x axis) which is color coded as red, blue and green for biological, cellular and molecular function processes respectively.

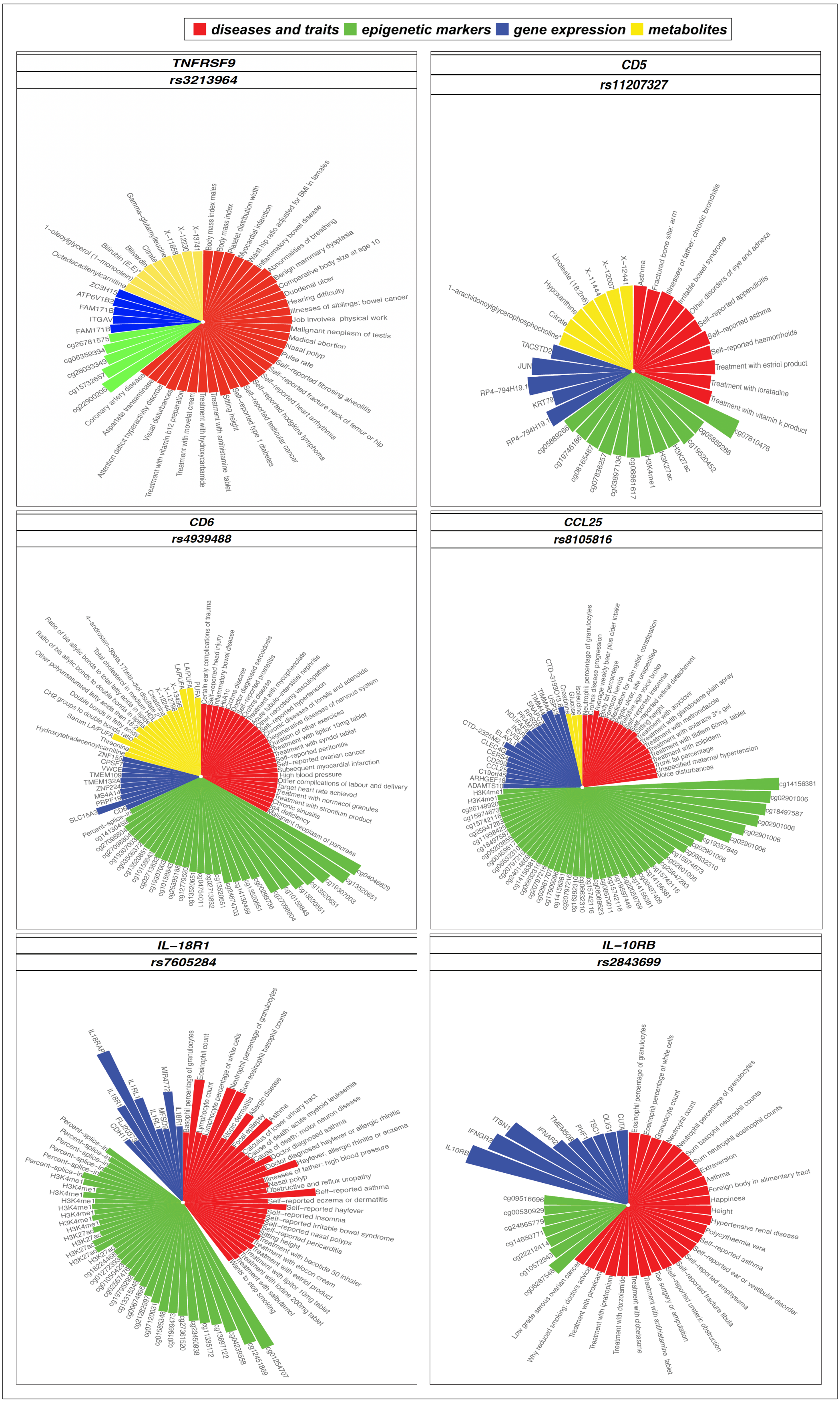

Figure S6: Circular barplots summarizing traits associated with male-specific pQTLs
The length of the bars denotes the strength of associations (P-values).

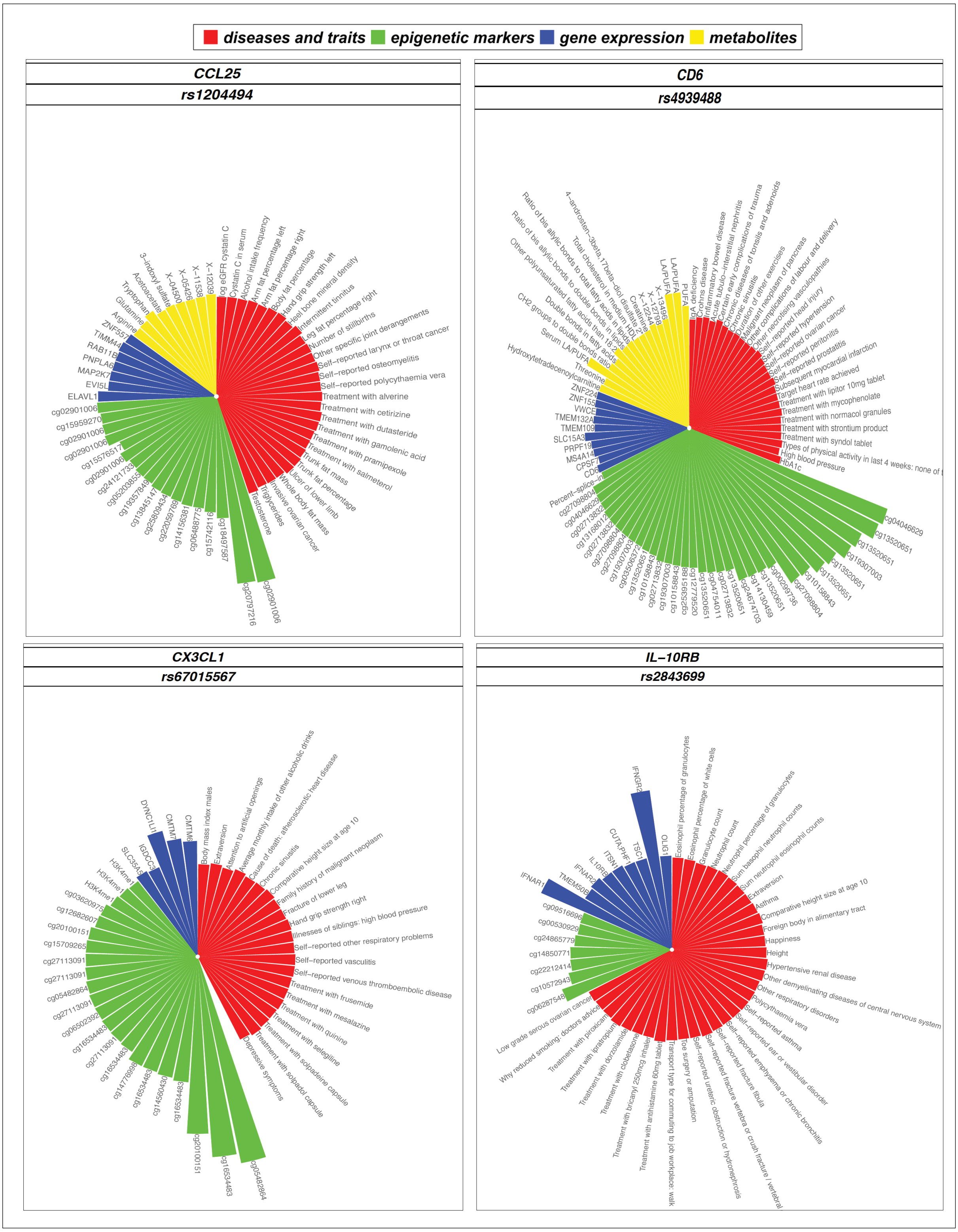

Figure S7A: Circular barplots summarizing traits associated with genome-wide significant pQTLs in females.

The length of the bars denotes the strength of associations (P-values). pQTL variants with cross-referenced association with all diseases & traits, epigenetic markers, gene expression and metabolites are plotted

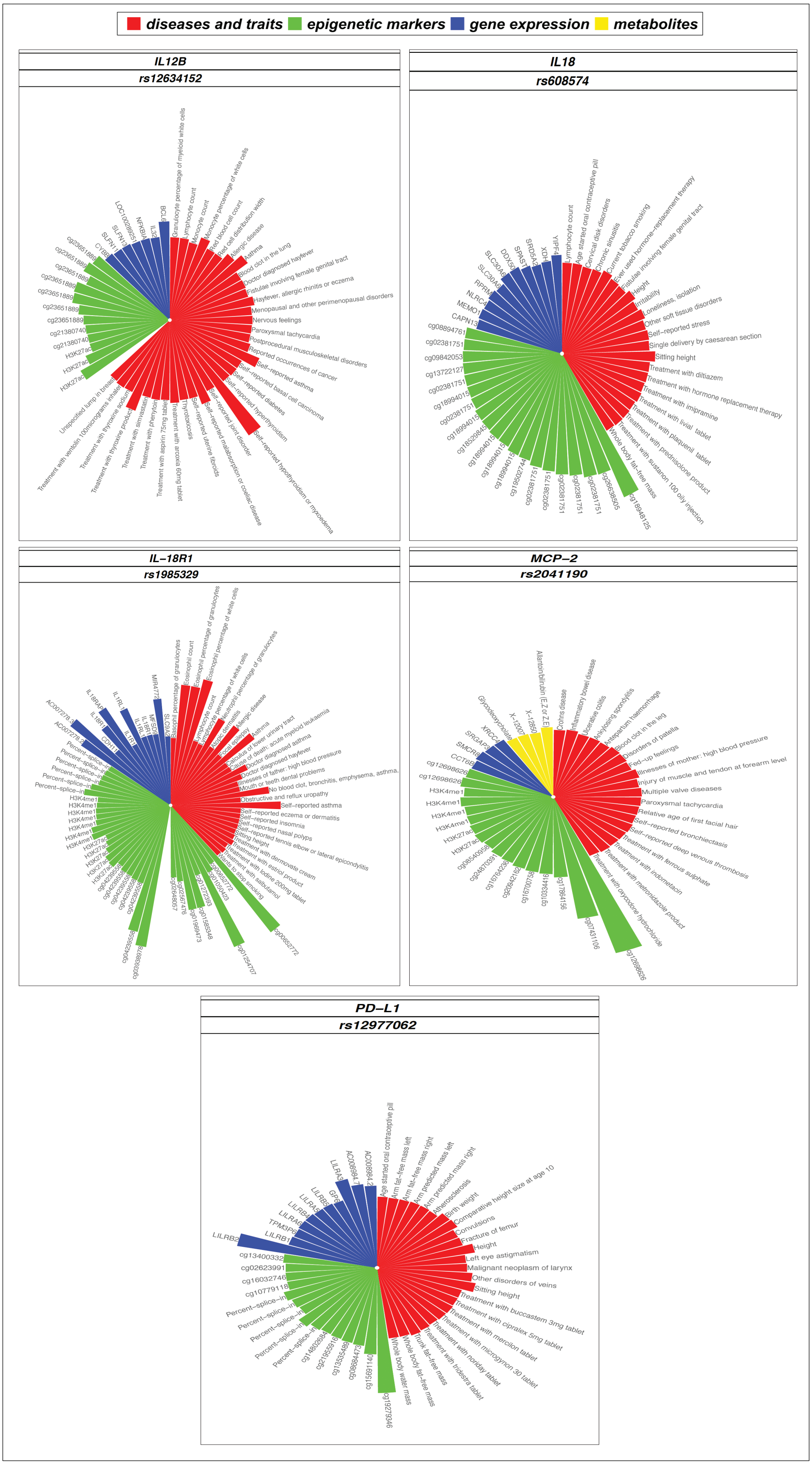

Figure S7B: Circular barplots summarizing traits associated with genome-wide significant pQTLs in females
